# Progressive Local Accumulation of Self-Assembled Nanoreactors in a Hydrogel Matrix through Repetitive Injections of ATP

**DOI:** 10.1021/jacs.1c13504

**Published:** 2022-01-21

**Authors:** Rui Chen, Krishnendu Das, Maria A. Cardona, Luca Gabrielli, Leonard J. Prins

**Affiliations:** Department of Chemical Sciences, University of Padova, Padova, 35131, Italy

## Abstract

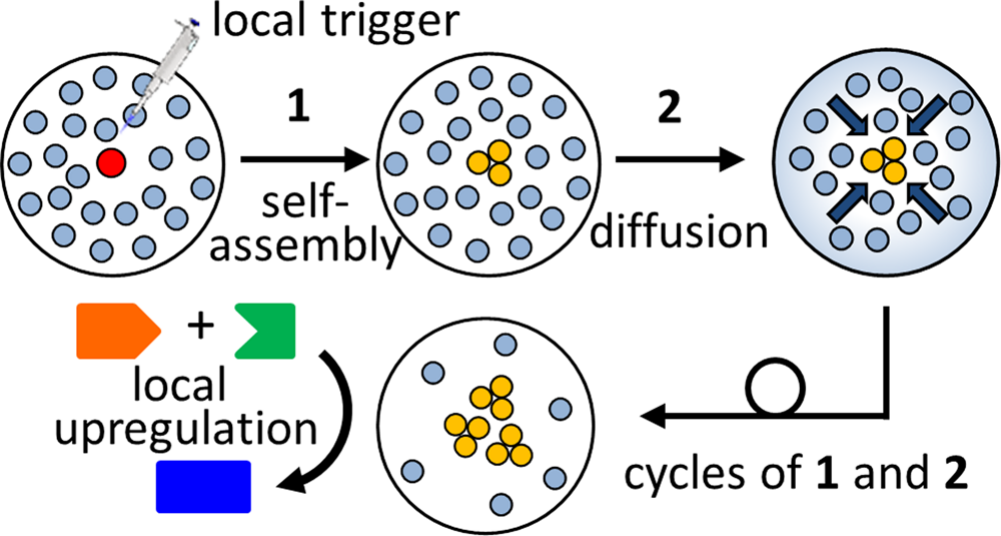

Cellular functions are regulated with high spatial control through the local activation of chemical processes in a complex inhomogeneous matrix. The development of synthetic macroscopic systems with a similar capacity allows fundamental studies aimed at understanding the relationship between local molecular events and the emergence of functional properties at the macroscopic level. Here, we show that a kinetically stable inhomogeneous hydrogel matrix is spontaneously formed upon the local injection of ATP. Locally, ATP templates the self-assembly of amphiphiles into large nanoreactors with a much lower diffusion rate compared to unassembled amphiphiles. The local depletion of unassembled amphiphiles near the injection point installs a concentration gradient along which unassembled amphiphiles diffuse from the surroundings to the center. This allows for a progressive local accumulation of self-assembled nanoreactors in the matrix upon repetitive cycles of ATP injection separated by time intervals during which diffusion of unassembled amphiphiles takes place. Contrary to the homogeneous matrix containing the same components, in the inhomogeneous matrix the local upregulation of a chemical reaction occurs. Depending on the way the same amount of injected ATP is administered to the hydrogel matrix different macroscopic distributions of nanoreactors are obtained, which affect the location in the matrix where the chemical reaction is upregulated.

## Introduction

The awareness that properties associated with life, such as motility, growth, replication, and adaptation, arise from a nonequilibrium state of matter has led to a strong interest in synthetic nonequilibrium systems that may lay the basis for the development of materials with life-like properties.^1,2^ In the cell, the nonequilibrium nature of life manifests itself at both the molecular and macroscopic level by the chemically fueled operation of the biological molecular machinery^3−6^ and the inhomogeneous distribution of molecular components within the cytosol, respectively.^7,8^ At both levels, nonequilibrium systems rely on the energy-fueled population of high energy states that subsequently release the stored potential energy to perform work.^9^ At the molecular level such states represent high-energy molecular conformations or molecular assemblies,^10^ whereas at the macroscopic level they represent an inhomogeneous distribution of components resulting in concentration gradients.^11−13^ In the cytoplasm, proteins are either locally produced and activated or are subject to a postsynthesis transport to specific cellular locations.^14,15^ Spatially controlled enzymatic activity installs concentration gradients in the cytosol which play an important role in regulating biological functions such as the positioning of the FtsZ protein ring—responsible for cell division—in the exact middle of the cell^16,17^ and the search and capture of kinetochores—protein complexes at the middle of each chromosome—by microtubules during cell division.^18^ Reaction-diffusion systems are currently extensively being explored in a synthetic context with the scope of understanding how properties such as chemotaxis, motility, and pattern formation arise from concentration gradients.^19−27^

**Figure 1:**
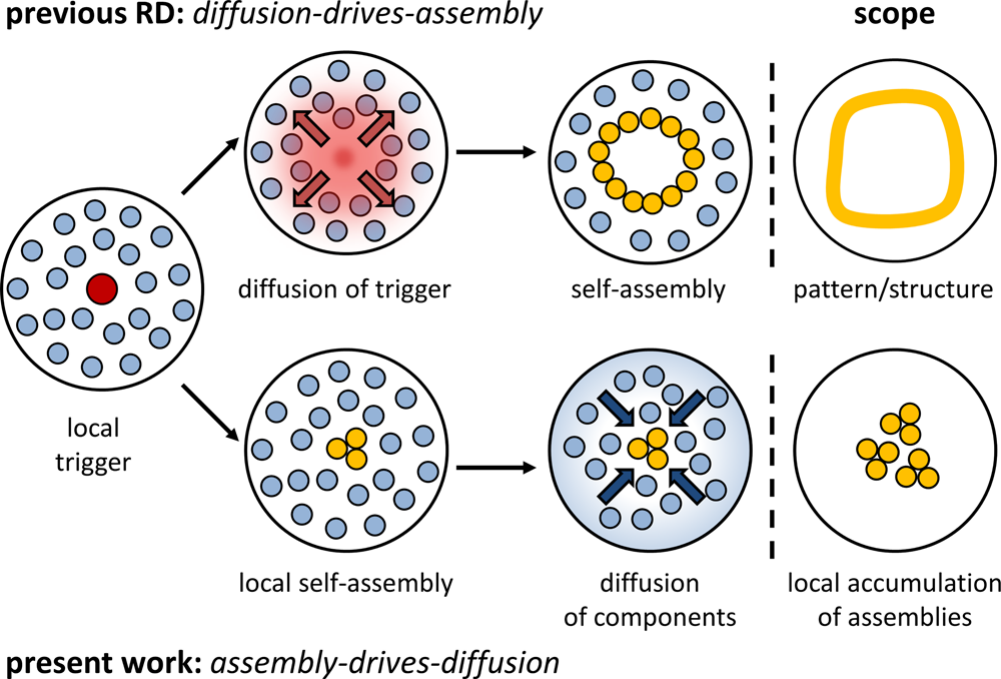
Instalment of concentration gradients in a hydrogel matrix upon the local application of a trigger. In previous work, diffusion of the trigger in the matrix led to the spatially controlled activation of a self-assembly process (top). Here, we show that the local activation of a self-assembly process by the trigger causes the diffusion of molecules embedded in the matrix leading toward a local accumulation of assemblies (bottom).

Whereas significant advances have been made in understanding how chemical energy can be used to populate molecular kinetically stable states,^28−32^ the chemically controlled population of macroscopic high-energy states has received far less attention.^33,34^ Very recent examples include the mechanisorption driven accumulation of ring-like molecules on a MOF surface and the active transport of substrates from solution to polymer beads.^35,36^ A key step in the development of macroscopically activated systems is the availability of methodology that provides spatial control over structure formation.^13,37^ A promising direction is provided by reaction-diffusion systems in which diffusion of a locally applied trigger (e.g., molecules,^33,38^ acid,^39,40^ redox^41,42^) through a hydrogel matrix activates structure formation in the matrix (Figure 1, top). Structure formation occurs either through the trigger-activated reaction between molecules that were already embedded in the matrix or through the direct reaction of the trigger with embedded molecules. This approach has predominantly been used to activate the formation of a second hydrogel in a pre-existing hydrogel matrix with the purpose of creating shapes and patterns or locally altering the material properties.^33,38−42^ However, for the purpose of populating a macroscopic kinetically stable state such a diffusion-followed-by-reaction scheme is less useful, because it does not permit the progressive population of such a state through repetitive additions of trigger–similar to what happens at the molecular level. Here, we show that this goal can be achieved by inverting the order of events, that is, by implementing a reaction-followed-by-diffusion scheme. Exploiting a hydrogel matrix, we show that the addition of a chemical trigger locally activates a templated self-assembly processes which locally depletes the concentration of unassembled molecules. Consequently, molecules present in the matrix spontaneously diffuse toward the addition point (Figure 1, bottom). These molecules can then be trapped in the assembled state upon the addition of an additional batch of the template. Repetitive cycles consisting of template addition and diffusion thus leads to a progressive local accumulation of assemblies. It will be shown that this macroscopic kinetically stable state has an enhanced capacity to upregulate a chemical reaction compared to a matrix in which the same components are homogeneously distributed.

## Results and Discussion

**Figure 2:**
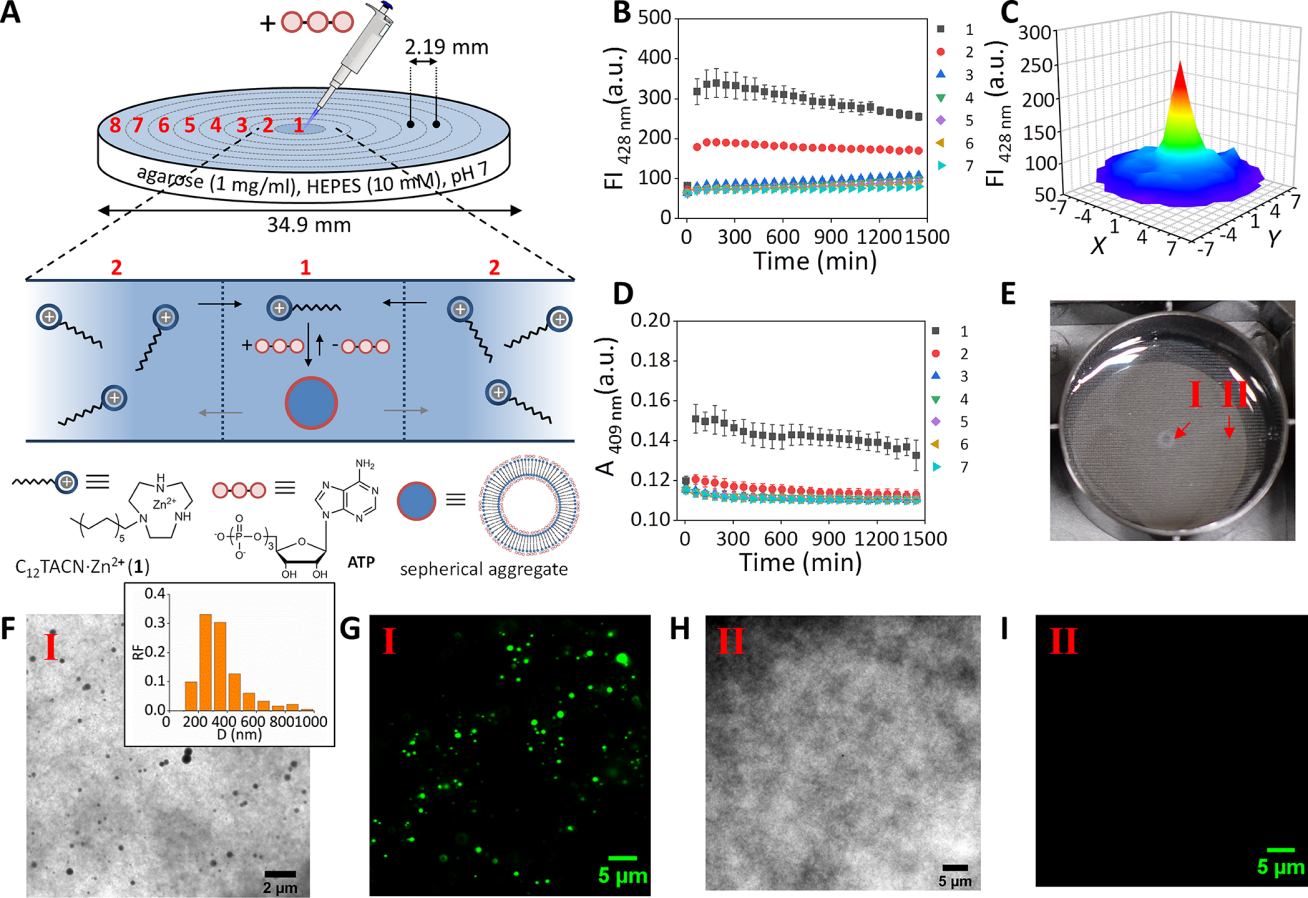
(a) Schematic representation of the templated assembly diffusion system studied in this work that relies on the local activation of the ATP-templated self-assembly of **1** (C_12_TACN·Zn^2+^) resulting in formation of spherical assemblies. The external position 8 was excluded to avoid border effects in the data analysis. The black arrows denote the relative fast diffusion of unassembled **1**, and the gray arrows denote the slow diffusion of the assemblies. (b) Changes in the fluorescence intensity of 1,6-diphenyl-1,3,5-hexatriene (DPH) at 428 nm of positions 1–7 as a function of time after 1 μL ATP (1 mM) was injected in the gel center. The increase in the period 0–200 min has been separately measured at a higher reading frequency (Figure S3). (c) 3D map of the fluorescence intensities of the entire gel at *t* = 1445 min after 1 μL of ATP (1 mM) was injected in the gel center. (d) Changes in the absorbance at 409 nm as a function of time after 1 μL of ATP (1 mM) was injected in the gel center. In this experiment the absorbance originates from the turbidity of the formed aggregates. The absorbance at 409 nm was measured because this wavelength was used in the chemical reactivity studies (Figure 5) to monitor the formation of hydrazone **C**. (e) Photograph of the gel after 1 μL of ATP (1 mM) was injected in the gel center at *t* = 300 min. (f) TEM image of a gel sample taken from area **I** in Figure 2e. The inset shows the relative frequency (RF) of the assemblies with a given diameter (D). (g) LSCM image of a gel sample taken from area **I** in Figure 2e. (h) TEM image of a gel sample taken from area **II** in Figure 2e. (i) LSCM image of a gel sample taken from area **II** in Figure 2e. General gel compositions and experimental conditions: [agarose] = 1 mg/mL, [HEPES] = 5 mM, pH 7.0, [**1**] = 100 μM, *T* = 25 °C. For fluorescence studies: [DPH] = 2.5 μM, λ_ex_/λ_em_ = 355/428 nm, slits = 5/10 nm (ex/em), gain = 100. Each point is the average of three experiments. Error bars indicate the standard deviation.

The design of chemical systems at the macroscopic level implies that also the kinetics of mass transport has to be taken into consideration. The formation of a macroscopic inhomogeneous state is facilitated in a matrix in which mass transport is controlled by diffusion. This is the case in the cytosol, which has a high viscosity as a result of macromolecular crowding.^43,44^ Here, we show that, in the absence of convection, a locally triggered molecular self-assembly process can become the source for an inhomogeneous distribution of matter at the macroscopic level. According to the Stokes–Einstein equation the diffusion coefficient of a species is inversely proportional to its diameter.^45^ This implies that the self-assembly of small molecules in large structures is accompanied by a significant decrease in diffusion rate. We reasoned that the difference in diffusion coefficient between unassembled and assembled state could lead to the spontaneous local accumulation of molecules in the matrix. The locally triggered templated self-assembly of large structures would lead to the local depletion of unassembled molecules resulting in a concentration gradient along which additional unassembled molecules would diffuse from the surroundings to the reaction center (black arrows in Figure 2a). An opposite concentration gradient would be installed for the assemblies, but since their diffusion occurs at a much lower rate, the diffusion of assemblies from the center to the surrounding would take much more time (gray arrows in Figure 2a). The result would be the formation of a kinetically stable inhomogeneous matrix characterized by a local presence of assemblies, but with a homogeneous distribution of unassembled molecules. A new injection of template would then capture the unassembled molecules that had diffused to the center and cycle-after-cycle this would lead to the progressive local accumulation of assemblies in the matrix.

This scenario bears to a certain extent conceptual resemblance to pattern-forming precipitation reactions.^46,47^ The self-organization of structures and patterns, such as chemical gardens^22,48^ and Liesegang structures,^49,50^ originates from local precipitation because of supersaturation, which drives diffusion by installing concentration gradients in the matrix.^51,52^ The structures are made up of inorganic materials, and the driving force for their formation is precipitation or phase separation. Yet, as a difference, in this study we demonstrate that the self-assembly of large purely organic structures—which remain homogeneously dissolved in the matrix—can also act as a driving force for the installment of concentration gradients. In addition, rather than the formation of macroscopic patterns, the scope of this study is to locally accumulate functional structures through repetitive self-assembly diffusion cycles.

An example of a self-assembly process characterized by a strong change in size—which may span up to orders of magnitude—is the self-assembly of amphiphilic molecules. We have previously shown that ATP can template the self-assembly of metalloamphiphile C_12_TACN·Zn^2+^ (**1**) in nanosized assemblies (*d* ≈ 100 nm) at a concentration far below the critical micellar concentration (CMC) of **1** (30 μM vs 5 mM, respectively).^53−56^ Efficient templation is attributed to a combination of enthalpic factors—originating from coordination bonds between the phosphates and the 1,4,7-triazacyclononane (TACN)·Zn^2+^ headgroup of **1**—and entropic factors—originating from the displacement of multiple counteranions with a single multivalent one.^57^

Hydrogels are very attractive matrices for creating a macroscopic inhomogeneous state since mass transport through convection is prevented by the presence of the polymer network. Indeed, in a previous study we have shown that continuous local UV-irradiation of a hydrogel containing nanoparticles and light-responsive molecules leads to the installment of a macroscopic nonequilibrium steady state characterized by persistent concentration gradients of the light-responsive molecules.^58^

Agarose gel (1 mg mL^–1^, buffered at pH 7.0) was prepared containing **1** at a concentration (100 μM) that is well below the CMC and the hydrophobic fluorescent dye 1,6-diphenyl-1,3,5-hexatriene (DPH, 2.5 μM), which is a reporter molecule for assembly formation. Control experiments revealed that agarose did not interfere with the ATP-templated self-assembly of **1** at the concentrations used (Figure S2). Gels were prepared in six-well microtiter plates to permit spatially controlled measurements of the fluorescence intensity using multispot analysis (Section 2, SI). A tiny volume of a concentrated adenosine triphosphate (ATP)-stock solution (1 μL, 1 mM) was injected in the central position 1 of the gel, and the fluorescence intensity of the entire gel was monitored as a function of time (Figure 2a). Upon injection we observed a strong increase in fluorescence intensity in the center (Figure 2b and Figure 2c). The fluorescence intensity reached a maximum after around 100 min, after which a very slow decrease in intensity was observed over time (ΔFI_24h_ = −30%). Importantly, no increase in fluorescence intensity was observed in other areas of the well. Local templated self-assembly was also evident from an increase in turbidity (Figure 2d) and could be visibly detected by an increased opacity in the center of the gel (Figure 2e) and was confirmed by transmission electron microscopy (TEM) (Figure 2f, 2h and Figure S12) and laser scanning confocal microscopy (LSCM) (Figure 2g, 2i and Figure S15). The analysis by TEM revealed that assemblies with dimensions in the range from 200 to 400 nm had exclusively formed in the area in which fluorescence and absorbance was detected.

The observation that surprised us was the high kinetic stability of the gel containing locally templated assemblies. This is coherent with the anticipated low diffusion rate of the large templated assemblies, but it is also reflects on the affinity of ATP for the assemblies. Indeed, dissociation of ATP from the assemblies and subsequent diffusion through the matrix is an alternative pathway for returning to a homogeneous matrix. Yet, the affinity of ATP for the assemblies is so high that the concentration of free ATP is very low and, consequently, also the concentration gradient that determines the rate of ATP-diffusion. However, it is not absent, which explains the slow decrease in fluorescence intensity over the time course of the experiment. In agreement with this explanation, the injection of adenosine diphosphate (ADP) (1 μL, 5.0 mM), which is a templating agent with lower affinity,^59^ resulted also in the local formation of assemblies, but, according to fluorescence measurements, in lower quantities and with a limited lifetime in the order of 7 h (Figure S4c). The injection of AMP (1 μL, 7.5 mM) did not induce any effect even though it was injected at a much higher concentration (Figure S4d).

**Figure 3:**
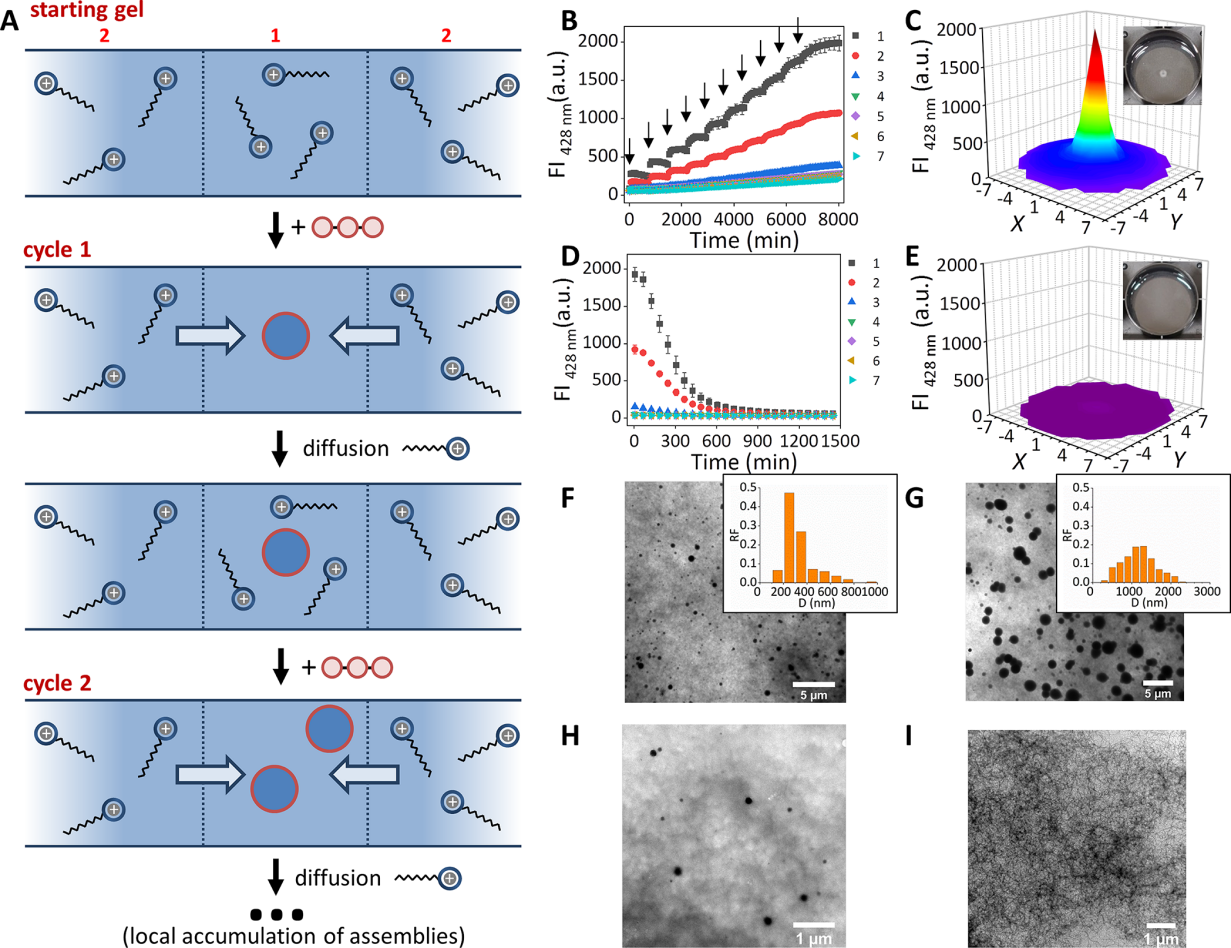
(a) Schematic representation of the templated assembly diffusion process leading to the local accumulation of assemblies upon repetitive cycles of ATP-injection followed by diffusion. Blue arrows indicate the diffusion of **1** from the surrounding to the gel center. (b) Changes in the fluorescence intensity at 428 nm as a function of time for positions 1–7 upon 10 injections of 1 μL of ATP (1 mM) in position 1 separated by 720 min intervals. The arrows indicate the time at which ATP was injected. (c) 3D map of the fluorescence intensity of the entire gel at *t* = 8015 min after 10 injections of 1 μL ATP (1 mM) in the gel center and the corresponding photo. (d) Changes in the fluorescence intensity at 428 nm as a function of time after 1 μL of alkaline phosphatase (10 KU) was injected in position 1 at the end of the experiment shown in Figure 3b. (e) 3D map of the fluorescence intensity of the entire gel at the end of the experiment shown in Figure 3d the corresponding photo. (f) TEM image of a gel sample (position 1) after the first 1 μL ATP (1 mM) aliquot was injected at *t* = 720 min. The inset shows the relative frequency (RF) of the assemblies with a given diameter (*D*). (g) TEM image of a gel sample (position 1) after addition of the 10th aliquot of 1 μL of ATP (1 mM) at *t* = 8015 min. The inset shows the relative frequency (RF) of the assemblies with a given diameter (*D*). (h) TEM image of a gel sample in which 1 μL of alkaline phosphatase (10 KU) was injected in position 1 at the end of the experiment shown in Figure 3d after 9 h. (i) TEM image of a gel sample in which 1 μL of alkaline phosphatase (10 KU) was injected in position 1 at the end of the experiment shown in Figure 3d after 24 h. General gel compositions and experimental conditions: [agarose] = 1 mg/mL, [HEPES] = 5 mM, pH 7.0, [**1**] = 100 μM, *T* = 25 °C. For fluorescence studies: [DPH] = 2.5 μM, λ_ex_/λ_em_ = 355 nm/428 nm, slits = 5/10 nm (ex/em), gain = 100. Each point is the average of three experiments. Error bars indicate the standard deviation.

According to our hypothesis, the local injection of ATP results in a gel that locally contains assemblies but has a homogeneous distribution of unassembled **1**. Indeed, calculation of the diffusion rate of unassembled **1** using the measured diffusion coefficient in the gel matrix (4.3 × 10^–6^ cm^2^ s^–1^) (Figure S21) showed that diffusion of unassembled **1** from position 1 to 2 (2.19 mm) requires around 1.6 h, which is far shorter than the observed lifetime for ATP-templated assemblies in position 1. The formation of a kinetically stable inhomogeneous gel state implies that potential energy is stored in the system on the macroscopic level. We reasoned that we could further populate this state—implying an increased storage of potential energy—by repetitive cycles of ATP addition followed by diffusion (Figure 3a). Each addition of ATP would trap locally available unassembled **1** in assemblies, but the local concentration of unassembled **1** would be restored by diffusion from the surroundings. Cycle after cycle this would lead to an accumulation of assemblies in the center. It is of interest to note that this process—repetitive provision of pulses of chemical energy—bears a strong resemblance to the strategy used to populate high-energy states of molecular machines at the molecular level.^60^ Experimentally, we subjected a gel to 10 injections of a 1 mM stock solution of ATP at intervals of 12 h to ensure sufficient time for equilibration of unassembled **1**. After each addition we observed a rise in both fluorescence intensity and turbidity indicating the formation of additional assemblies (Figure 3b and Figure 3c and Figure S9f), but always exclusively in the center. Comparison of TEM images taken after the 1st and 10th injection showed the increase in assembly concentration after multiple injections and confirmed the progressive population of the kinetically stable state (Figure 3f and 3g and Figure S13a and S13b).

The formation of the kinetically stable gel state is possible because of the low concentration of free ATP in the system. Consequently, ATP diffuses very slowly from the center to the outer regions of the matrix, which permits stability of the ATP-templated assemblies in the center. Considering that adenosine monophosphate (AMP) is unable to install a kinetically stable gel state (Figure S4d), we argued that the enzymatic dephosphorylation of ATP would allow the matrix to relax back to a uniform distribution of molecules.^61−64^ This hypothesis was confirmed by the observation that injection of the enzyme alkaline phosphatase, an enzyme that converts ATP in adenine and 3 inorganic phosphates, Pi, in the center of the gel that had been subjected to 10 ATP injections resulting in the complete disappearance of fluorescent intensity over a time course of several hours and the complete disappearance of turbidity (Figure 3d and Figure 3e). The disappearance of the assemblies was confirmed by TEM analysis (Figure 3h and 3i and Figure S13c and S13d).

**Figure 4:**
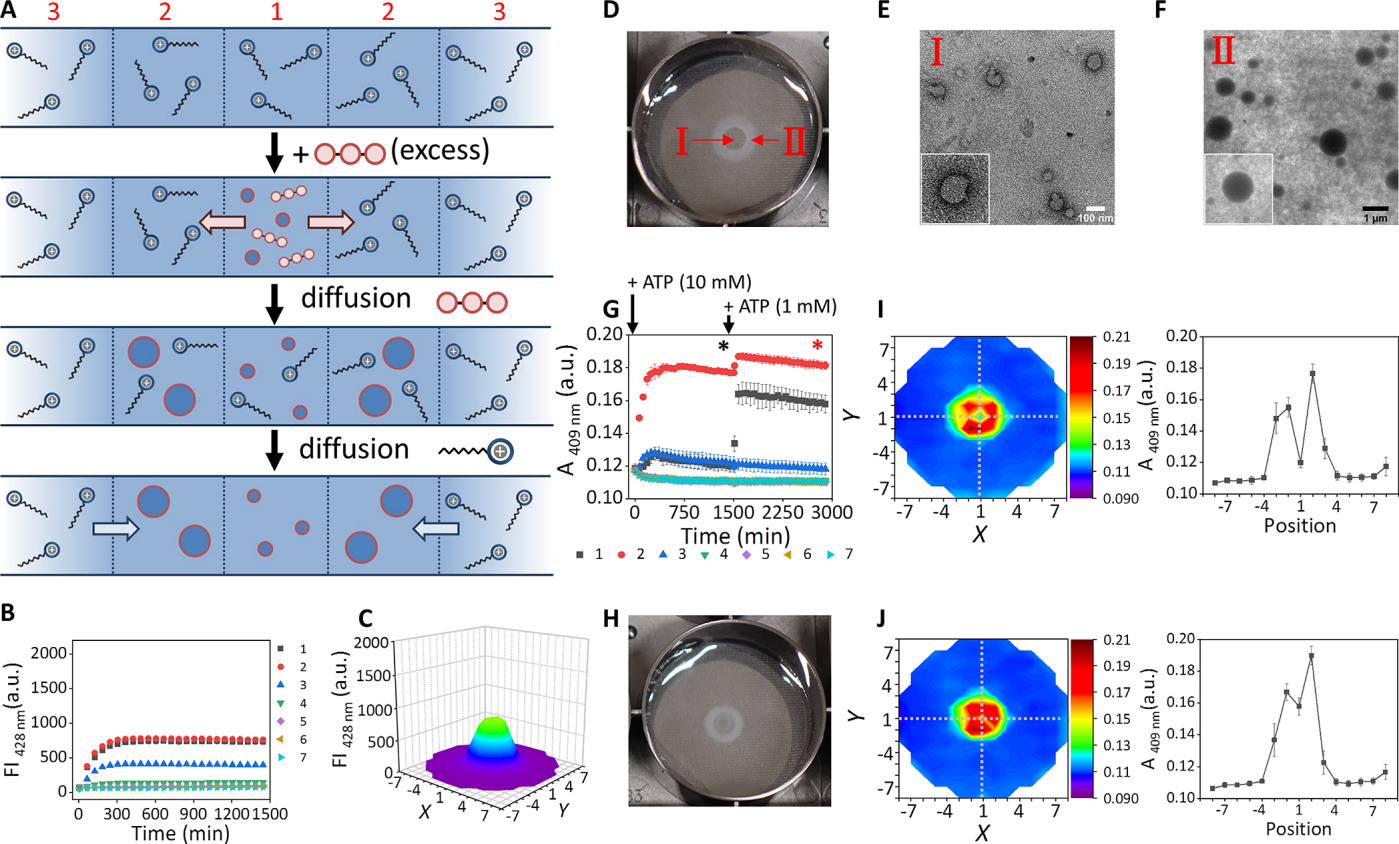
(a) Schematic representation that illustrates how a single injection of a large quantity of ATP (1 μL, 10 mM) induces assemblies of different size at different locations. (b) Changes in the fluorescence intensity at 428 nm as a function of time for positions 1–7 after injection of ATP (1 μL, 10 mM) in position 1. (c) 3D map of the fluorescence intensity of the entire gel at *t* = 1445 min after injection of ATP (1 μL, 10 mM) in position 1. (d) Photograph of the gel at *t* = 300 min after ATP (1 μL, 10 mM) was added to position 1. (e) TEM images of the assemblies formed in the transparent area of the gel (area **I** in Figure 4d). (f) TEM images of the assemblies formed in the opaque area of the gel (area **II** in Figure 4d). (g) Changes in the absorbance at 409 nm as a function of time after ATP (1 μL, 10 mM) was injected in position 1, followed by the injection of an additional injection of ATP (1 μL, 1 mM) after 1505 min. (h) Photograph of the gel taken 1390 min after an additional amount of ATP (1 μL, 1 mM) was injected in position 1 of the gel of Figure 4d. (i) 2D color contour of the absorbance of the entire gel at *t* = 1505 min after injection of ATP (1 μL, 10 mM) in position 1 (corresponding to the black asterisk in Figure 4g). Changes in absorbance at 409 nm for positions 1–7 corresponding to the cross sections indicated with dashed lines in the 2D map. (j) 2D color contour of the absorbance of the entire gel taken 1390 min after an additional amount of ATP (1 μL, 1 mM) was injected in position 1 of the gel (corresponding to the orange asterisk in Figure 4g). Changes in absorbance at 409 nm for positions 1–7 corresponding to the cross sections indicated with dashed lines in the 2D map. General gel compositions and experimental conditions: [agarose] = 1 mg/mL, [HEPES] = 5 mM, pH 7.0, [**1**] = 100 μM, *T* = 25 °C. For fluorescence studies: [DPH] = 2.5 μM, λ_ex_/λ_em_ = 355 nm/428 nm, slits = 5/10 nm (ex/em), gain = 100. Each point is the average of three experiments. Error bars indicate the standard deviation.

Further evidence for the formation of a macroscopic kinetically stable state and its gradual population upon repetitive injections of ATP was obtained from an experiment in which the final amount of ATP was administered to the gel in a single injection (1 μL, 10 mM, Figure 4a–c). Surprisingly, rather than the opaque center observed after multiple 1 mM ATP-injections, turbidity measurements revealed the presence of an opaque ring with a diameter of around 8 mm and an inner transparent area (Figure 4d). Also this gel had a high kinetic stability and hardly any detectable changes were observed over the time course of 24 h (Figure 4b, c, and g). TEM images from samples taken from the transparent center and the opaque ring revealed in both cases the presence of assemblies, but of very different size (Figures 4e and 4f). Assemblies taken from the turbid ring had the same dimensions as the assemblies observed previously in the center of the gel that was injected with small amounts of ATP (Figure 4f and Figure S14b). On the other hand, much smaller assemblies with dimensions in the order of 100 nm were detected in the transparent center (Figure 4e and Figure S14a). The formation of the ring structure can be rationalized by considering that previously reported solution studies had shown that the size of ATP-templated assemblies of **1** depended strongly on the ratio **1**/ATP.^53,54^ An excess of ATP to **1** resulted in the formation of extended aggregates composed of tiny spherical substructures with dimensions similar to those observed for assemblies in the transparent inner area of the ring. On the other hand, smaller amounts of ATP led to spherical assemblies with a diameter compatible with the assemblies present in the opaque area. Based on this information, we postulate that the injection of a large quantity of ATP traps all locally available **1** in small assemblies. Contrary to what happens in solution,^54^ these assemblies are prevented from aggregation in the gel matrix. The excess of ATP diffuses away and, at reduced concentrations, templates the formation of larger assemblies distant from the injection point. The scattering of light from these larger sized assemblies creates the visual effect of the ring structure. Coherent with this explanation, the injection of ATP in a concentration range from 1 to 25 mM in gels containing the same concentration of **1** resulted in the formation of the larger assemblies—evidenced by ring structures of increased diameter—at increasing distances from the center (Figure S5).

The observation that gels with an identical chemical composition can have different macroscopic distributions of molecules that depend on the way the same amount of ATP is administered to the gel (10 injections of 1 mM vs a single injection of 10 mM) unequivocally demonstrates that these gels represent different macroscopic states of matter. Importantly, a single injection of ATP (even up to 25 mM) never led to the fluorescence intensity observed after 10 repetitive injections of a 1 mM ATP stock solution (Figure 3b and Figure S6a and S6b). This observation underlines the importance of the diffusion of unassembled **1** as a prerequisite for the local accumulation of assemblies. Equilibration of unassembled **1** occurs also in the gel state containing the ring structure obtained after a single 10 mM injection of ATP. Consequently, the additional injection of 1 mM ATP in that gel resulted in the additional formation of large assemblies in the center as indicated by the absorbance increase from the turbidity of position 1 (Figure 4g–j and Figure S7). This shows that unassembled **1** had again become available in the center of the gel because of diffusion.

**Figure 5:**
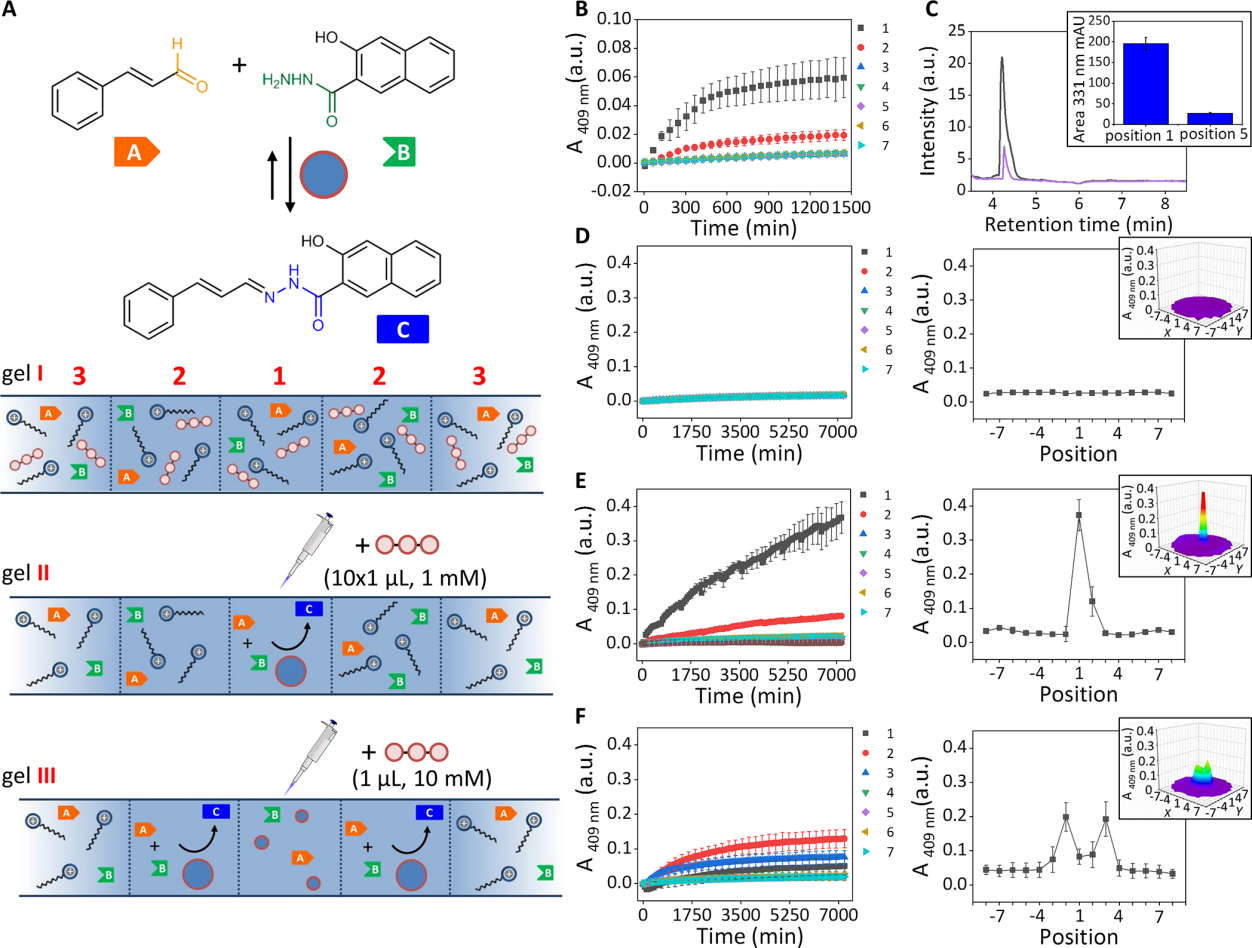
(a) Formation of hydrazone **C** upon reaction between *trans*-cinnamaldehyde **A** and 3-hydroxy-2-napthoic hydrazide **B** accelerated by ATP-templated assemblies of **1**. Schematic representation that shows local upregulation of hydrazone formation in gels in which the same amount of ATP (10 mM) is homogeneously distributed (**I**), injected in small aliquots (**II**, 10 × 1 μL, 1 mM) or injected in a single aliquot (**III**, 1 μL, 10 mM). (b) Changes in the absorbance at 409 nm for positions 1–7 as a function of time after ATP (1 μL, 1 mM) was injected in position 1. (c) UPLC chromatograms of samples taken at 24 h from the gel described in Figure 5b (black trace: position 1, purple trace: position 5). The histogram in the inset reports the integration of the area of the peak corresponding to **C** for position 1 and 5. (d) Changes in the absorbance at 409 nm as a function of time when ATP (10 μL, 1 mM) was homogeneously distributed in the gel. Changes in absorbance at 409 nm for positions 1–7 corresponding to the cross sections. The inset gives a 3D map of the absorbance of the entire gel for *t* = 7205 min. (e) Changes in the absorbance at 409 nm for positions 1–7 as a function of time after 10 injections of ATP (1 μL, 1 mM) in position 1 separated with time intervals of 720 min. Changes in absorbance at 409 nm for positions 1–7 corresponding to the cross sections. The inset gives a 3D map of the absorbance of the entire gel for *t* = 7175 min. (f) Changes in the absorbance at 409 nm as a function of time after 1 μL ATP (10 mM) was added in gel center. Changes in absorbance at 409 nm for positions 1–7 corresponding to the cross sections. The inset gives a 3D map of the absorbance of the entire gel for *t* = 7205 min. General gel compositions and experimental conditions: [agarose] = 1 mg/mL, [*trans*-cinnamaldehyde] = 20 μM, [3-hydroxy-2-napthoic hydrazide] = 20 μM, [HEPES] = 5 mM, pH 7.0, [**1**] = 100 μM, *T* = 25 °C. Each point is the average of three experiments. Error bars indicate the standard deviation. For experiments b, d, e, f, the absorbance values were corrected for turbidity (see Supporting Information section 9).

An inhomogeneous distribution of matter in the matrix becomes purposeful if it has a functional property that is not active in the homogeneous matrix. We have previously shown in solution studies that ATP-templated assemblies of **1** can strongly accelerate the formation of hydrazone **C** between *trans*-cinnamaldehyde **A** and 3-hydroxy-2-napthoic hydrazide **B** (Figure 5a).^53^ The observed 25-fold rate acceleration was attributed to an increase in the effective concentration as a result of the uptake of the hydrophobic reactants in the hydrophobic domain of the assemblies. The absence of an increase in absorbance at 409 nm in a gel containing **A** (20 μM), **B** (20 μM), and amphiphile **1** (100 μM) indeed confirmed the slow rate of the background reaction in the hydrogel matrix (Figure S8a). On the other hand, the injection of 1 μL of a stock solution of ATP (1 mM)—the condition for the formation of large assemblies in the center—resulted in an immediate strong increase in absorbance in the center, which, after correction from the contribution by scattering, could be attributed to the formation of **C** (Figure 5b and Figure S9a and S9b). The local formation of **C** was independently verified by UPLC by measuring samples taken at various positions in the gel (Figure 5c and Figure S24). The comparison of the integration of the peak corresponding to **C** in samples taken at positions 1 and 5 revealed that after 24 h a significant larger amount of product **C** had formed in position 1. The concentration of **C** in position 1 was quantified at around 2 μM based on a previously reported calibration curve.^53^ The injection of the same amount of either ADP or AMP did not result in any significant formation of **C** (Figure S8b and Figure S8c).

After having confirmed that the injection of ATP locally creates the conditions for product formation, we were interested to find out how the upregulation of reactivity would take place in different gel states. We followed the formation of **C** in three different gels (**I**–**III**) with the same chemical composition, but different distributions of assemblies. Gel **I** is the thermodynamically stable state in which ATP (10 μL × 1 mM) is distributed homogeneously, whereas gels **II** and **III** are inhomogeneous states obtained respectively from 10 injections of ATP (1 mM) or 1 injection of ATP (10 mM) (Figure 5a). The absence of any significant increase in absorbance in gel **I** over the time course of 120 h showed that the thermodynamic state is unable to upregulate the chemical reaction (Figure 5d). On the contrary, a strong upregulation was observed both for gels **II** and **III**, but with strong differences regarding the location where the major increase in product formation occurred. In gel **II** product formation occurred exclusively in the center (Figure 5e), whereas in gel **III**—featuring the ring structure—product had predominantly formed in the opaque area where large assemblies were present (Figure 5f). The overall absorbance increases in gels **II** and **III** were nearly the same indicating the formation of the same quantity of product on the macroscopic level (Figure S11). These results show that the procedure for administrating ATP to the gel provides control over the location where the reaction is upregulated.

## Conclusion

In conclusion, we have shown that a thermodynamically controlled self-assembly process at the molecular level can become the source for the spontaneous formation of a macroscopic kinetically stable state with an inhomogeneous distribution of organic molecules present in the matrix. The formation of this state relies essentially on two features: first, a large difference in size exists between the assembled and unassembled state, and second, the affinity of the template for the assembly is so high that a concentration gradient for free template is practically absent. It is shown that the macroscopic kinetically stable state can be progressively populated through repetitive cycles consisting of ATP-addition followed by diffusion of unassembled building blocks toward the center. The inhomogeneous matrix is more effective in upregulating a chemical reaction compared to a matrix in which the same components are distributed homogeneously. The approach is based on generic criteria that match many supramolecular processes, which suggests that this approach could find wide applicability for the formation of macroscopic systems in which functional hotspots can be created to serve as nodes in complex macroscopic reaction-diffusion networks.

## Supporting Information Available

Materials and instrumentation, procedure for gel preparation and characterization (DOSY NMR, TEM, LSCM, UV–vis, fluorescence measurements, UPLC), data analysis, photographs of gels, supplementary figures, and supplementary references (PDF)
