# Supplementary material for: Progressive Local Accumulation of Self-Assembled Nanoreactors in a Hydrogel Matrix through Repetitive Injections of ATP
Source: J Am Chem Soc. 2022 Jan 21;144(4):2010–8. doi: 10.1021/jacs.1c13504 (PMC8815075; doi:10.1021/jacs.1c13504)
Supplement: Supplementary file 1 — ja1c13504_si_001.pdf [file ja1c13504_si_001.pdf]

SUPPORTING INFORMATION

Progressive Local Accumulation of Self-Assembled Nanoreactors in a Hydrogel Matrix Through Repetitive Injections of ATP

Rui Chen, Krishnendu Das, Maria A. Cardona, Luca Gabrielli, Leonard J. Prins*

Department of Chemical Sciences, University of Padova, Via Marzolo 1, 35131 Padova, Italy

Table of Contents

1. Materials and instrumentation 2

2. Gel preparation and data analysis 4

3. Study of the critical aggregation concentration (*cac*) of the metallo-surfactant C_{12}TACN·Zn^{2+} and the ATP-templated self-assembly of C_{12}TACN·Zn^{2+} in gel 6

4. Study of the initial rate of ATP-templated self-assembly of 1 in gel 7

6. Formation of different macroscopic kinetically stable states as a function of the concentration of injected ATP 9

7. Diffusion of unassembled 1 11

8. Upregulation of hydrazone C formation upon injection of ADP and AMP 14

9. Measurement of the absorbance increase in gels resulting from hydrazone formation 15

10. Total amount of hydrazone C formed in gels to which the same amount of ATP was administered in different fashion 17

11. Transmission electron microscopy (TEM) images 18

12. Laser scanning confocal microscopy (LSCM) images 21

13. Diffusion coefficients obtained from DOSY 23

14. UV-Vis spectra of compounds A, B and C in gel 27

15. UPLC analysis 28

16. Supplemental References 29

1. Materials and instrumentation

Materials

All commercially available reagents were purchased from Merck unless mentioned otherwise and used as received. Low electroendosmosis (EEO) agarose was used. NMR spectra were recorded using commercially available deuterated solvents, while all the UV-Vis and fluorescence measurements were carried out using deionized water filtered by a Milli-Q water purifier (Millipore). The 6-well glass bottom plates were purchased from Cellvis.

Adenosine 5'-triphosphate (ATP) disodium salt hydrate, adenosine 5'-diphosphate (ADP) sodium salt, and adenosine 5'-monophosphate (AMP) sodium salt stock solutions were prepared in MilliQ water by weight and the exact concentration was calculated by UV-Vis spectroscopy using the molar extinction coefficient: ε_{259} (ATP, ADP, AMP) = 15400 M^{-1} cm^{-1}.

The *trans*-cinnamaldehyde and the hydrazide 3-hydroxy-2-napthoic hydrazide stock solutions were prepared by weight in acetonitrile at the required concentration, typically 20 mM, fresh before use. The concentration of *trans*-cinnamaldehyde was confirmed by UV-Vis spectroscopy using the molar extinction coefficient: ε_{284} = 25300 M^{-1} cm^{-1}.

$Zn(NO_3)_2$ was analytical grade product. The concentration of the metal ion stock solution was determined by ICP (inductively coupled plasma) analysis.

The buffer 4-(2-hydroxyethyl)-1-piperazineethanesulfonic acid (HEPES, Sigma) was used without further purification.

The stock solution of the fluorophore 1,6-diphenyl-1,3,5-hexatriene (DPH) was prepared by weight in THF (tetrahydrofuran).

The enzyme alkaline phosphatase solution was diluted in 1 mL MilliQ water to give a concentration of 10 KU/mL or 1 KU/mL. The solution of 1 KU/mL was further diluted to obtain a concentration of 100 U to be used for lower enzyme concentrations.

The synthesis and characterization of C_{12}TACN has been reported[1]. The stock solution of C_{12}TACN was prepared by weight in MilliQ water to give a concentration about 5 mM.

Hydrazone **C** 3-hydroxy-N'-((1E,2E)-3-phenylallylidene)-2-naphthohydrazide was synthesized using a published procedure[2].

Instrumentation

pH Measurements: The pH of buffer solutions was determined at room temperature using a Metrohm-632 pH meter equipped with a Ag/AgCl/KCl reference electrode and calibrated with standard buffer solutions at pH 7.00.

UV-Vis Measurements: UV-Vis spectra and kinetics were recorded on a Varian Cary50 spectrophotometer equipped with thermostatted multiple cell holders and on a TECAN M1000 PRO plate reader.

Fluorescence Spectroscopy: Fluorescence measurements were recorded on a Varian Cary Eclipse fluorescence spectrophotometer and a TECAN M1000 PRO plate reader.

UPLC Analysis: The UPLC analysis were performed on an Agilent Technologies 1290 Infinity equipped with a DAD detector and a Quadrupole LC/MS. Column: Agilent Zorbax SB-C3 Rapid Resolution HT 3.0 × 100 mm 1.8 micron.

TEM Analysis: TEM images were recorded on a Jeol 300 PX electron microscope. One drop of sample was placed on the sample grid for 1 minute. For staining purposes (Figure 3d in the main text), it was then placed on a drop of uranyl acetate (2%) for 30 s. The solvent was evaporated before the stained grid was imaged. TEM images were elaborated using the software ImageJ.

LSCM Analysis: Confocal images were taken using a laser scanning confocal microscope (BX51WI-FV300-Olympus) coupled to a frequency doubled Ti:Sapphire femtosecond laser at 400 nm, 76 MHz (VerdiV5Mira900-F Coherent). The laser beam was scanned on a 40x40 μM sample area with a 512x512 resolution, using a 60x water immersion objective (UPLSAPO60xW-Olympus).

Photographs: Photographs and video clips were taken with a Sony DSC-RX100M5.

NMR Analysis: DOSY spectra were recorded using a Bruker AV III 500 NMR spectrometer. Chemical shifts (δ) are reported in ppm using residual solvent value as internal reference. The pulse sequence used for Diffusion Ordered Spectroscopy (DOSY) experiments is 1edbpgp2s. The data were all analyzed and exported using the Dynamics Center 2.5.6.b1 software package (Bruker Biospin).

2. Gel preparation and data analysis

Gel preparation

For general fluorescence experiments: For the preparation of 3 mL HEPES-buffered agarose gel containing C_{12}TACN, $Zn(NO_3)_2$, and DPH, 3 mg of agarose was weighted in a 4 ml vial and dissolved in 2778 µl of Milli-Q water using mild heating (closed vial). After all agarose had dissolved, the transparent homogeneous solution was left at room temperature to cool until 40 °C (approximately 3 minutes). Then 150 µL of a HEPES-stock solution (100 mM, final concentration 5 mM), 60 µL of a C_{12}TACN stock solution (5 mM, final concentration 100 µM), 9.2 µL of a $Zn(NO_3)_2$ stock solution (32.9 mM, final concentration 100 µM), and 3 µL of a DPH stock solution (2.5 mM, final concentration 2.5 µM) were added. After gentle shaking, the solution was quickly transferred to a 6-well glass bottom plates after which a sealing film was applied to avoid evaporation. A transparent gel was obtained after 1 h solidification at room temperature. Generally, the measurements and ATP injection (1 µL) in gel center were started 2 h after the preparation of gels.

Gels for absorbance measurements (containing $C_{12}TACN \cdot Zn^{2+}$, ATP and/or *trans*-cinnamaldehyde **A** and/or 3-hydroxy-2-napthoic hydrazide **B**) were prepared in the same way. The gelation concentration of agarose is about 0.1% (w/v). This gel density is sufficient to maintain the gel state.

Data analysis

Measurements were performed at 177 points of each well, distributed as shown in Figure S1. The external area of the well (1 mm from the border) was excluded to avoid border effects. Each data point was the average of 3 measurements at the same location.

The data points were grouped in 8 areas, corresponding to 8 positions from center to border (see color scheme in Figure S1 and general presentation in Figure 1a) with a distance between two adjacent positions of 2.19 mm. The external position 8 was excluded to avoid border effects in the data analysis.

Fluorescence measurements of gels containing a homogeneous concentration of DPH revealed that the gel slightly increased in thickness from the center to the border. To permit a correct quantitative comparison between the measurements carried out at the different positions 1 – 8 of each well, the measured values were corrected for the gel thickness.

Correction of the fluorescence intensities (DPH experiments) and absorbance values (kinetic experiments)

The different thickness across the gel in microplates affects the optical pathway and consequently the measured signal intensity. After grouping the data points in the 8 areas as described above, for each position a normalization factor was calculated relative to the initial value of position 7 (that is, referring to a gel in which components are homogeneously distributed and concentration gradients are absent). The obtained normalization factors were then used to correct for each position the measured fluorescence intensities/absorbance during the kinetics.

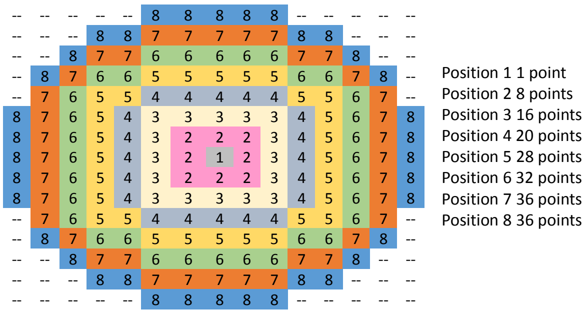

Figure S1. Multiple positions signal (177 points) was read for each well. The gel was grouped in 8 positions and the obtained value for each position was an average of multiple reads. The external position 8 was excluded to avoid border effects in the data analysis.

3. Study of the critical aggregation concentration (*cac*) of the metallo-surfactant $C_{12}TACN{\cdot}Zn^{2+}$ and the ATP-templated self-assembly of $C_{12}TACN{\cdot}Zn^{2+}$ in gel

To study the critical aggregation concentration (*cac*) of **1** ($C_{12}TACN{\cdot}Zn^{2+}$) in gel, a series of gels containing DPH (2.5 µM) and different amounts of **1** (0-1000 µM) were prepared and the fluorescence intensities were measured using the plate reader. In agreement with previously reported solution studies,[1] which had shown that the *cac* of **1** was higher than than 1000 µM, no significant fluorescence intensities were observed up to 1000 µM of **1**, indicating that agarose at 1 mg/ml does not induce assembly of **1** (Figure S2a). The ATP-templated self-assembly of **1** was studied in agarose by fluorescence measurements (plate reader) exploiting the uptake of the fluorescent probe DPH in the hydrophobic domain of the assemblies. Just as in the previous experiment, the concentration of **1** was gradually increased, but this time in the presence of ATP (50 µM). An identical profile was obtained as reported previously for the same study carried out in solution.[1] No significant change in critical aggregation concentration was detected. Furthermore, similar to the solution studies, at higher concentrations of **1** a decrease in fluorescence intensity was observed indicating that the amount of ATP was insufficient to stabilize assemblies. Importantly, though, it also confirms that agarose is unable to template assemblies. Altogether, these results show that the self-assembly process of ATP and **1** is not affected by agarose (Figure S2b).

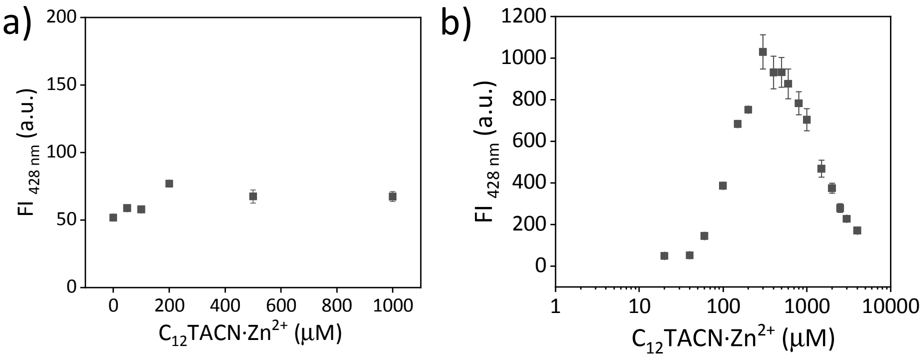

Figure S2. a) Study of the self-assembly of **1** in gel by fluorescence measurements. **b)** ATP templated self-assembly of **1** in agarose. Gel compositions: [agarose] = 1 mg/mL, [HEPES] = 5 mM, [**1**] = 0 – 1000 µM for **a** and 0 - 4000 µM for **b**, [DPH] = 2.5 µM and [ATP] = 50 µM for **b**. General experimental conditions: $\lambda_{ex}/\lambda_{em}$ = 355/428 nm, slit = 5/10 nm, gain = 100, pH 7, 25 °C. Each point is the average of three experiments. Error bars indicate the standard deviation.

4. Study of the initial rate of ATP-templated self-assembly of 1 in gel

To visualize the initial rate of the increase in fluorescence intensity for the experiment reported in Figure 2b was repeated taking recordings at shorter interval of 15 minutes in the initial period 0 – 200 minutes (Figure S3). The fluorescence intensity reached the maximum after around 100 minutes after ATP injection.

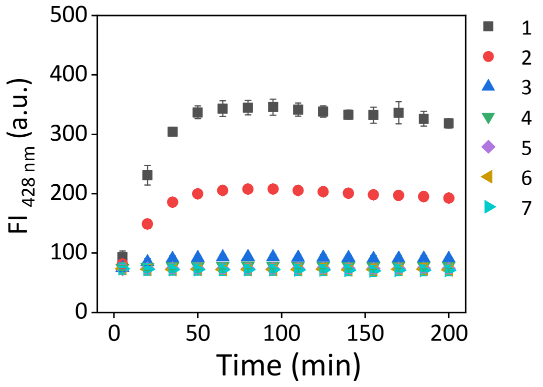

Figure S3. Changes in the fluorescence intensity of DPH at 428 nm of positions 1-7 as a function of time after 1 μL ATP (1 mM) was injected in the gel center. Gel composition and experimental condition: [agarose] = 1 mg/mL, [**1**] = 100 μM, [DPH] = 2.5 μM, λex/λem = 355/428 nm, slits = 5/10 nm (ex/em), gain = 100, [HEPES] = 5 mM, pH 7.0, T = 25 °C. Each point is the average of three experiments. Error bars indicate the standard deviation.

5. Templated self-assembly of 1 using ATP/ADP/AMP

To support the hypothesis that the high kinetic stability of the locally formed ATP-templated assemblies in gel is a result of the low dissociation rate of ATP from the assemblies, the same volume (1 µL) of stock solutions of ATP (2.5 mM or 5 mM), ADP (5 mM) and AMP (7.5 mM) were injected in the center of a gel containing **1** (100 µM) and DPH (2.5 µM). Upon the injection of ATP (either at 2.5 or 5 mM) the local formation of assemblies was observed, which were stable in time according to the constant fluorescence intensity (Figure S4a and S4b, respectively). For ADP, only a minor, transient increase in fluorescence intensity was observed (Figure S4c), whereas for AMP no increase in fluorescence intensity was observed at all (Figure S4d). These results show that ADP and AMP are not able to template stable assemblies of **1**.

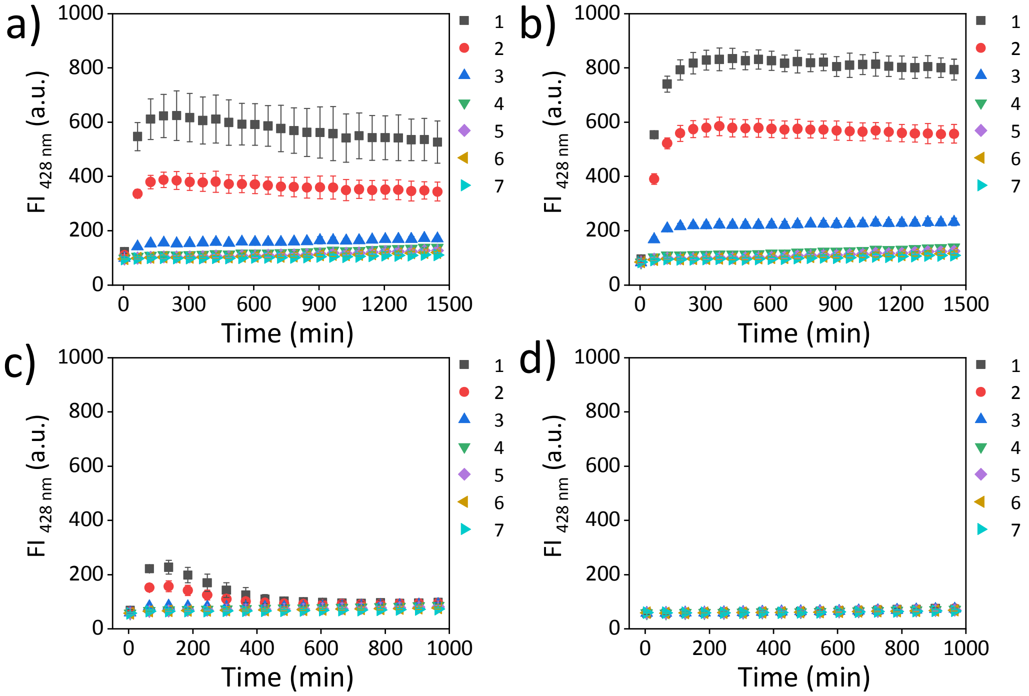

Figure S4. Fluorescence intensities at 428 nm of positions 1-7 as a function of time after 1 µL of nucleotide stock solution was injected in the gel center. **a)** 2.5 mM ATP, **b)** 5.0 mM ATP, **c)** 5.0 mM ADP, **d)** 7.5 mM AMP. Gel compositions: [agarose] = 1 mg/mL, [HEPES] = 5 mM, [**1**] = 100 µM, [DPH] = 2.5 µM. Experimental conditions: λex/λem = 355/428 nm, slit = 5/10 nm, gain = 100, pH 7, 25 °C. Each point is the average of three experiments. Error bars indicate the standard deviation.

6. Formation of different macroscopic kinetically stable states as a function of the concentration of injected ATP

The same volume of ATP(1 µL) from stock solutions at different concentrations (1 to 25 mM) was injected in the center of gels containing the same amount of **1** (100 µM) The turbidity from the larger sized assemblies creates the visual effect of the ring structure at increasing distances from the center (Figure S5a, photograph taken 1500 min after injection of ATP) Absorbance values at 409 nm were measured for all gels and plotted as a function of time for positions 1-7 (Figure S5b). 2D plots of the absorbance values of the gels at t=1500 min is provided in Figure S5c.

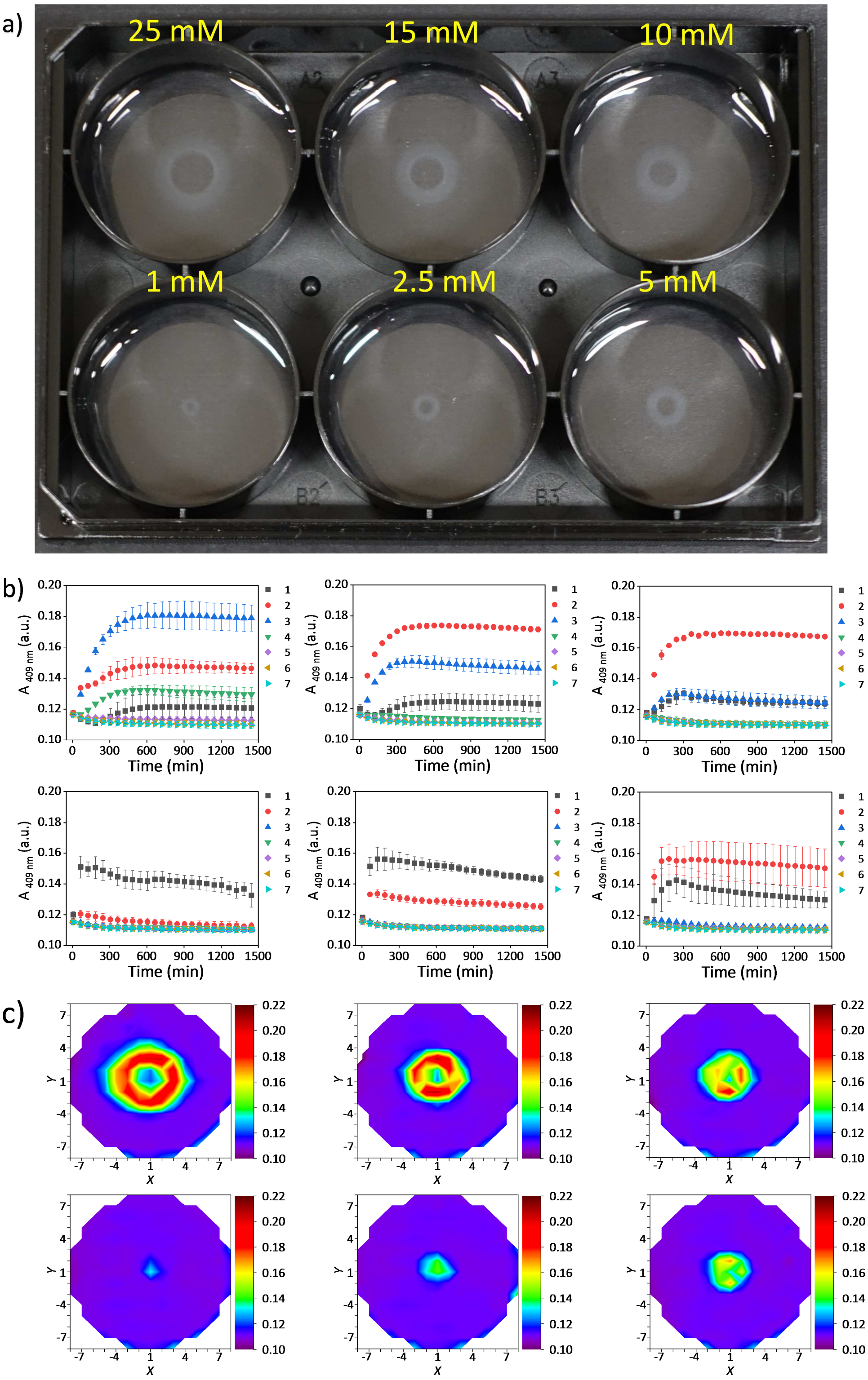

Figure S5. a) Photograph of the 6-well microplate containing gels with ring like structures obtained by injecting ATP at different concentrations (1-25 mM) in the center of the gel. The ATP concentration is indicated on the top of corresponding well. **b)** The absorbance traces of positions 1-7 of each gel at 409 nm as a function of time after 1 μL of ATP was injected. The order corresponds to the order in Figure S5a. **c)** The 2D color contour of the gels after the ATP injections for 24 h. The order corresponds to the arrangement of gels in Figure S5a. Experimental conditions: [agarose] = 1 mg/mL, [**1**] = 100 μM, [HEPES] = 5 mM, pH 7, 25 °C. Each point is the average of three experiments. Error bars indicate the standard deviation.

7. Diffusion of unassembled 1

To support the diffusion of unassembled **1** after locally ATP-templated assembly formation, initially 1 μL of ATP (25 mM, 15 mM) was injected in the center of buffered gel containing **1** (100 μM) and DPH (2.5 μM). After the fluorescence intensity had reached the maximum value, the gel was kept for around 24 hours and the presence of the ring structure was observed (Figure S6g). Next, additional ATP (1 μL, 1 mM) was injected in the (transparent) center of the gel and it was observed (Figure S6a and S6b) that the fluorescence intensities of positions 1 and 2 started to increase, but not the other positions. Simultaneously, an opaque spot formed in the transparent inner area of the ring. After an additional 24 h, another 1 μL of ATP (1 mM) was injected in the center of the same gel resulting in a further increase in fluorescence intensity of just position 1 and position 2, which reached the same value as measured for position 3 (obtained after the initial 25 mM ATP injection) (Figure S6a and S6b). Another smaller ring structure was observed inside the larger ring (formed from 25mM and 15 mM ATP injection) originating from the additional ATP (1 μL x 2, 1 mM) injections (Figure S6h). These observations indicate that after the formation of ATP-templated assemblies, diffusion of unassembled **1** takes place leading to a gel state with a homogeneous distribution of **1.**

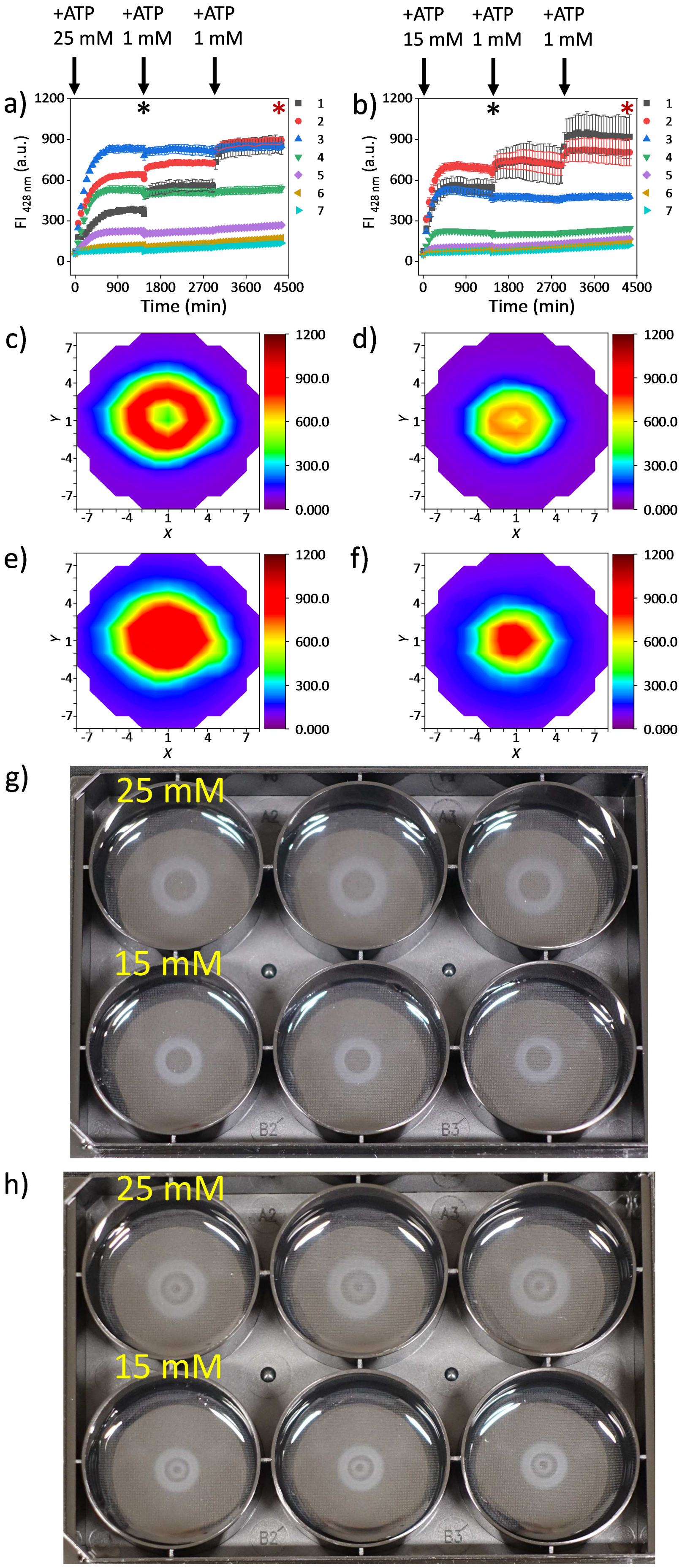

+ATP 25 mM
+ATP 1 mM
+ATP 1 mM
+ATP 15 mM
a)
b)
c)
d)
e)
f)
g)
h)
FI 428 nm (a.u.)
Time (min)
1
2
3
4
5
6
7
25 mM
15 mM

Figure S6. a), b) Fluorescence intensity at 428 nm as a function of time for positions 1-7 after the injection of 1 μL of an ATP stock solution (a: 25 mM, b: 15 mM) in the gel center, followed by two additional injections of ATP (1 μL, 1 mM) in position 1 separated by 24 h intervals. **c), d)** 2D color contour of the fluorescence intensity of the entire gel at t = 1445 min (indicated with a black asterisk in Figure S6a and Figure S6b. **e), f)** 2D color contour of the fluorescence intensities of the entire gel at t = 4345 min (indicated with a red asterisk in Figure S6a and Figure S6b. **g)** Photograph of the gel at t = 1445 min (black asterisk indicated) after ATP was added to position 1 (1 μL, 25 mM to the 3 wells on the top and 15 mM to the 3 wells at the bottom). **h)** Photograph of the gel taken 2900 min after additional amount of ATP (1 μL x 2, 1 mM) was injected in position 1 of the gel in Figure S6g (red asterisk indicated). Gel compositions: [agarose] = 1 mg/mL, [HEPES] = 5 mM, [**1**] = 100 μM, [DPH] = 2.5 μM. For fluorescence experiment conditions: λex/λem = 355/428 nm, slit = 5/10 nm, gain = 100, pH 7, 25 °C. Each point is the average of three experiments. Error bars indicate the standard deviation.

a)

b)

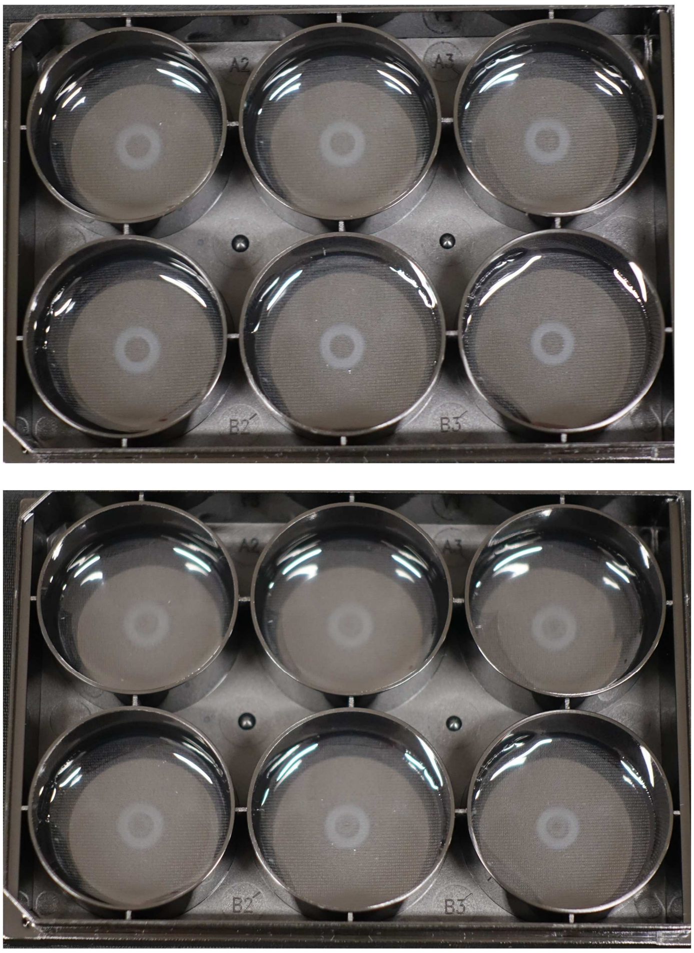

Figure S7. a) Photograph of gels taken at t = 6 hours after 1 μL of an ATP stock solution (10 mM) was injected in the gel center. The diameter of the formed rings is 8.2 ± 0.2 mm, and the diameter of the inside transparent area is 5.4 ± 0.4 mm. The values are the average of the 6 samples. **b)** Photograph of gels taken at t = 6 hours after an additional 1 μL of an ATP stock solution (1 mM) was injected to the gels shown in Figure S7a. Additional turbidity was observed in the center (diameter: 4.0 ± 0.1 mm). Gel compositions and experimental conditions: [agarose] = 1 mg/mL, [HEPES] = 5 mM, pH 7.0, [**1**] = 100 μM, T = 25 ^{o}C.

8. Upregulation of hydrazone C formation upon injection of ADP and AMP

The injection of either ADP or AMP (1 µL, 1 mM) did not result in any significant formation of hydrazone **C**, evidenced by the absence of an increase in absorbance (Figure S8).

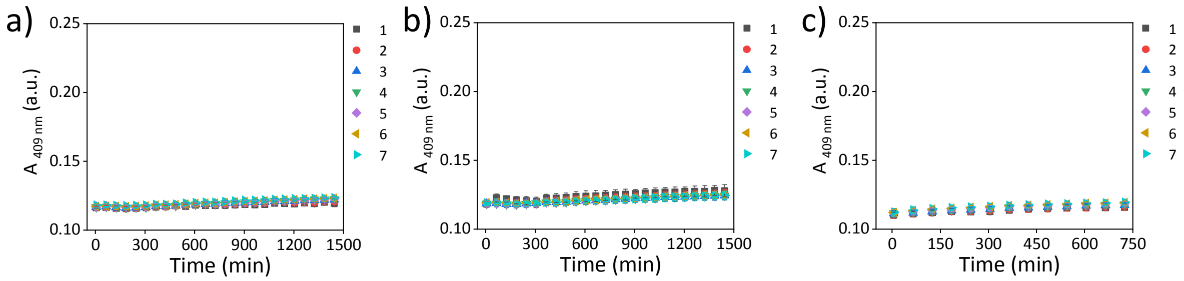

Figure S8. a) Absorbance at 409 nm as a function of time for positions 1-7 of the background reaction (no injection of any template). **b)** Absorbance at 409 nm as a function of time for positions 1-7 after 1 µL ADP (1.0 mM) was injected in the gel center. **c)** Absorbance at 409 nm as a function of time for positions 1-7 after 1 µL AMP (1.0 mM) was injected in the gel center. Gel compositions and experimental conditions: [agarose] = 1 mg/mL, [*trans*-cinnamaldehyde] = 20 µM, [3-hydroxy-2-napthoic hydrazide] = 20 µM, [HEPES] = 5 mM, pH 7.0, [**1**] = 100 µM, T = 25 °C. Each point is the average of three experiments. Error bars indicate the standard deviation.

9. Measurement of the absorbance increase in gels resulting from hydrazone formation

The data presented in Figure 5 of the manuscript correspond to the increase in absorbance resulting from the formation of hydrazone **C**, corrected for the increase in absorbance resulting from turbidity. The latter information was obtained from control experiments in which the same amount of template was added to a gel that did not contain reactants **A** and **B** (see Figure S9).

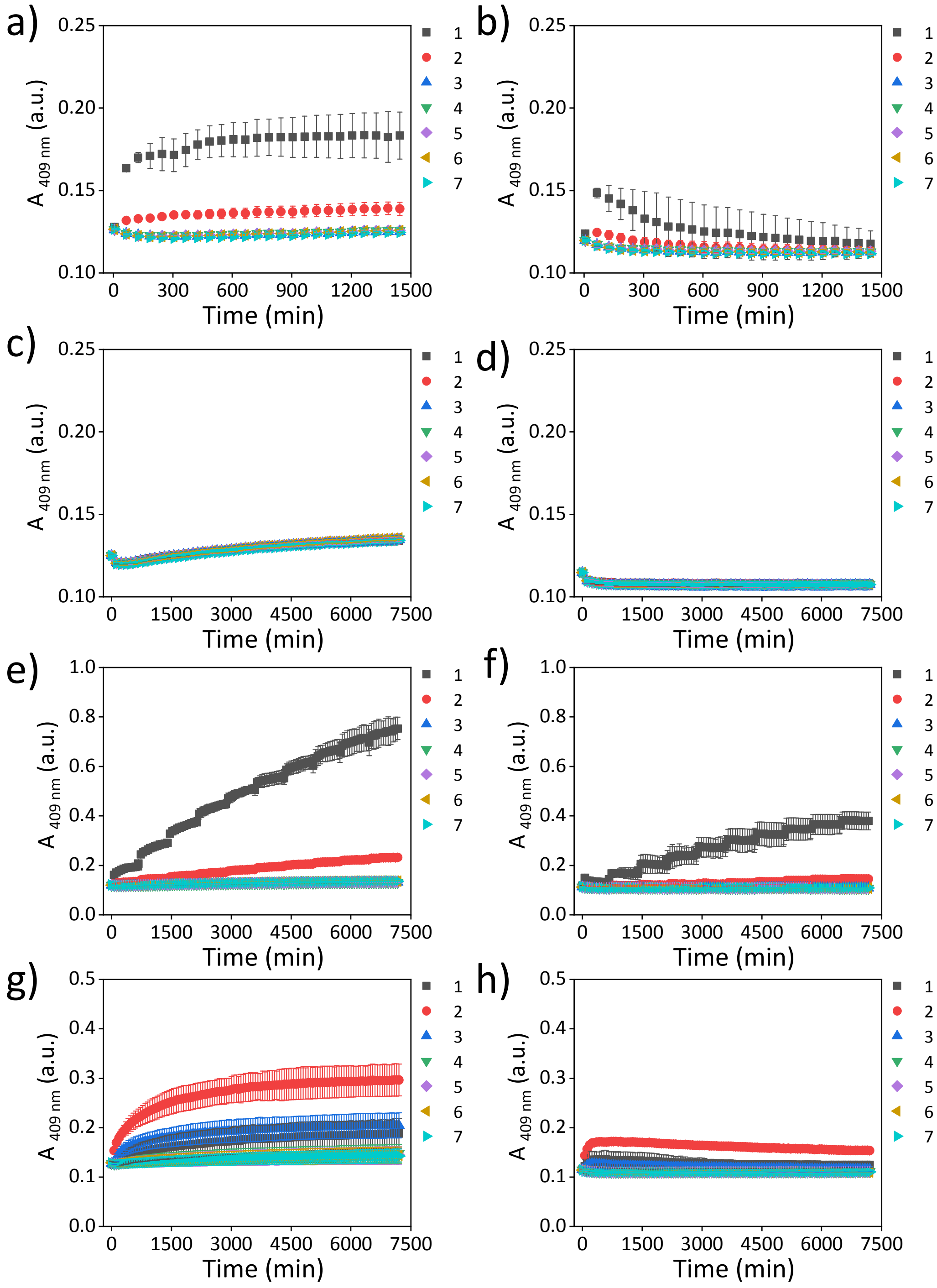

Figure S9. a) Absorbance at 409 nm as a function of time for positions 1-7 after 1 μL of an ATP stock solution (1 mM) was injected in the center of the gel containing reactants **A** and **B**. **b)** Absorbance at

409 nm as a function of time for positions 1-7 after 1 μL of an ATP stock solution (1 mM) was injected in the center of the gel in the absence of reactants **A** and **B**. **c)** Absorbance at 409 nm as a function of time for positions 1-7 when 10 μL of an ATP stock solution (1 mM) was added during the preparation of the gel - implying a homogeneous distribution of ATP in the gel - containing reactants **A** and **B**. **d)** Absorbance at 409 nm as a function of time for positions 1-7 when 10 μL of an ATP stock solution (1 mM) was added during the preparation of the gel – implying a homogeneous distribution of ATP in the gel – in the absence of reactants **A** and **B**. **e)** Absorbance at 409 nm as a function of time for positions 1-7 after 10 injections (1 μL x 10 injections) of an 1 mM ATP stock solution in the center of the gel containing reactants **A** and **B** separated by 720 mins intervals. **f)** Absorbance at 409 nm as a function of time for positions 1-7 after 10 injections (1 μL x 10 injections) of a 1 mM ATP stock solution in the center of the gel in the absence of reactants **A** and **B**. separated by 720 mins intervals. **g)** Absorbance at 409 nm as a function of time for positions 1-7 after 1 μL of an ATP stock solution (10 mM) was injected in the center of a gel containing reactants **A** and **B**. **h)** Absorbance at 409 nm as a function of time for positions 1-7 after 1 μL of an ATP stock solution (10 mM) was injected in the center of gel in the absence of reactants **A** and **B**. Gel compositions and experimental conditions: [agarose] = 1 mg/mL, [*trans*-cinnamaldehyde] = 20 μM, [3-hydroxy-2-napthoic] = 20 μM, [HEPES] = 5 mM, pH 7.0, [**1**] = 100 μM, T = 25 ^{o}C. Each point is the average of three experiments. Error bars indicate the standard deviation.

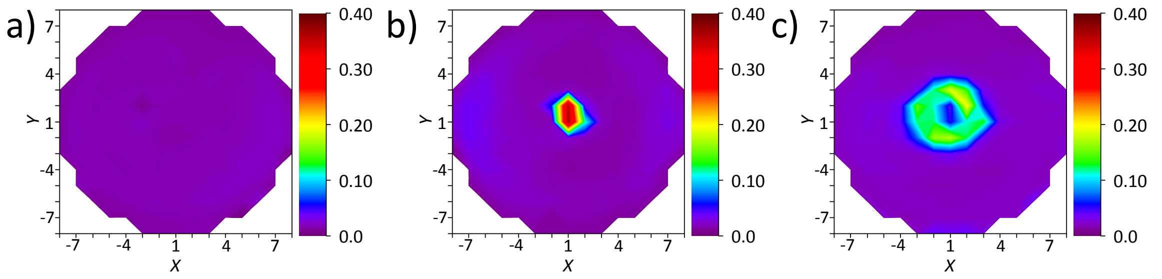

Figure S10. **a)** 2D color contour of the absorbance of the entire gel at t = 7205 min as described in the main text (Figure 5d). **b)** 2D color contour of the absorbance of the entire gel at t = 7175 min as described in the main text (Figure 5e). **c)** 2D color contour of the absorbance of the entire gel at t = 7205 min as described in the main text (Figure 5f).

10. Total amount of hydrazone C formed in gels to which the same amount of ATP was administered in different fashion

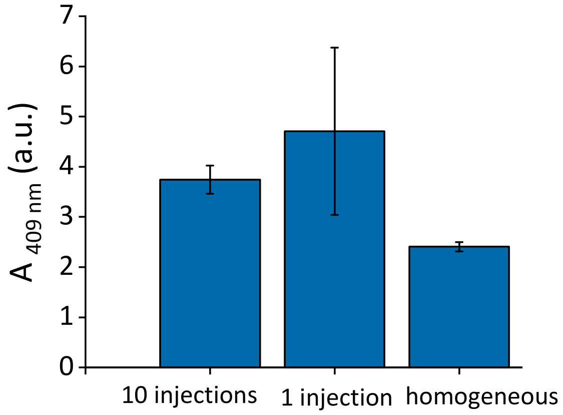

Figure S11. Total absorbance at 409 nm in the entire gel originating from the formation of hydrazone **C** obtained by summing the absorbance (weighted) in positions 1-7. The data for the 10 injections experiment corresponds to the value at t = 7175 min in Figure 5e. For the homogeneous and 1 injection experiments the values correspond to the data at t = 7205 min in Figure 5d and 5f in the main text. Each data is the average of three experiments. Error bars indicate the standard deviation.

11. Transmission electron microscopy (TEM) images

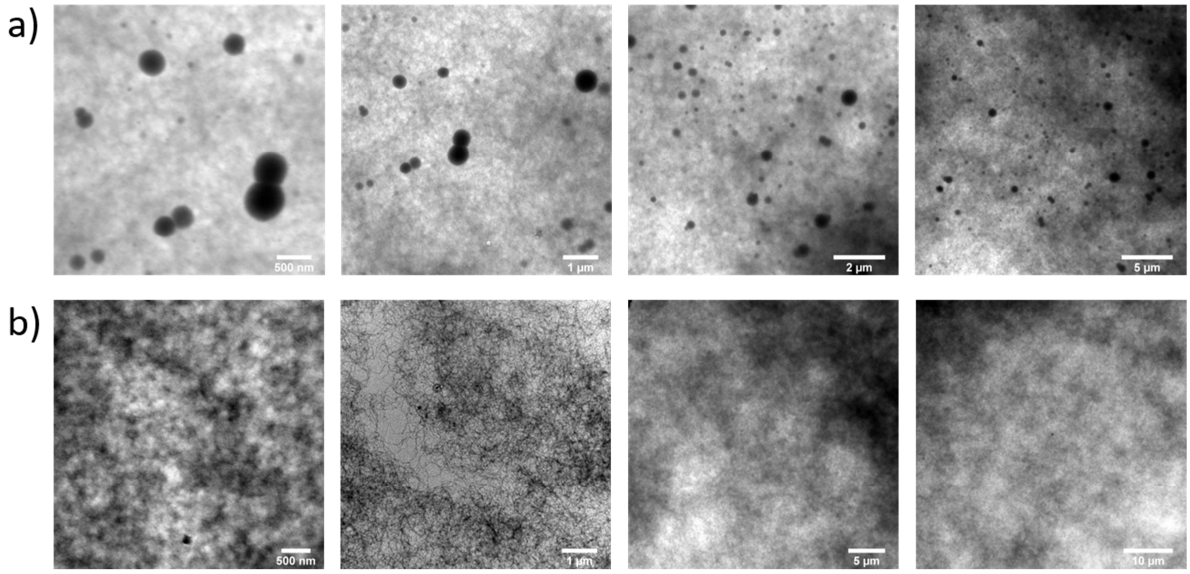

Figure S12. Additional TEM images of the gel to which 1 µL of ATP stock solution (1 mM) was injected in the center. **a)** Additional TEM images from the opaque area in the gel center after 1 µL of a stock solution of ATP (1 mM) was injected at t = 300 min as described in the main text (Figure 2f). **b)** Additional TEM images of the external transparent area from the gel to which 1 µL of a stock solution of ATP (1 mM) was injected in the gel center at t = 300 min as described in the main text (Figure 2h). Gel compositions and experimental conditions: [agarose] = 1 mg/mL, [HEPES] = 5 mM, pH 7.0, [**1**] = 100 µM, T = 25 °C.

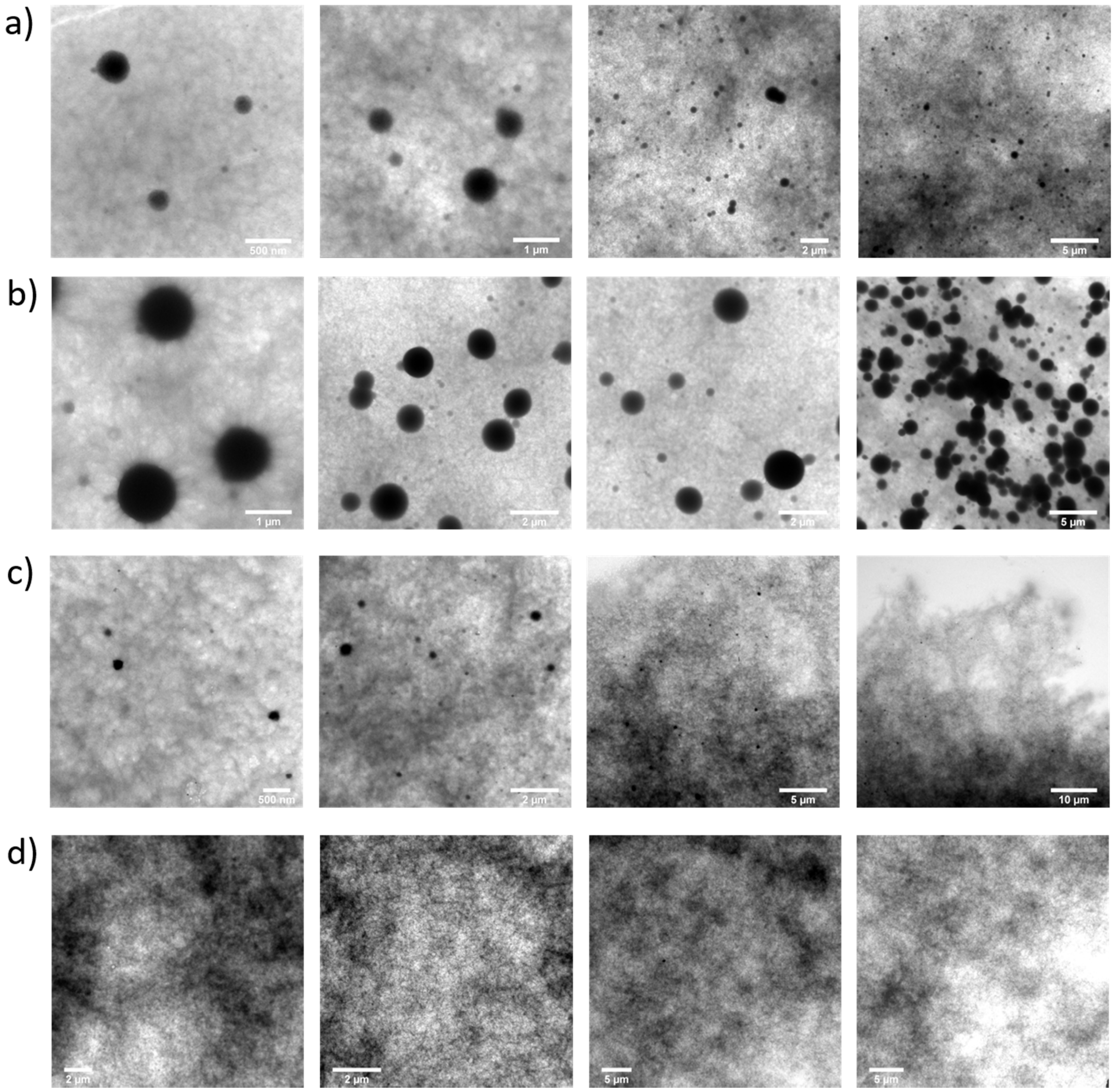

Figure S13. a) Additional TEM images of position 1 of a gel after the first 1 µL of a stock solution of ATP (1 mM) was injected at t = 720 min. **b)** Additional TEM images of position 1 of a gel after addition of the 10th aliquot (1 µL) of an ATP stock solution (1 mM) at t = 8015 min. **c)** Additional TEM images of the gel of Figure S13b to which 1 µL of alkaline phosphatase (10 KU) was injected in position 1 at t= 9 hr. **d)** Additional TEM images of the same gel as shown in Figure S13c 24 hours after injection of the enzyme. Gel compositions and experimental conditions: [agarose] = 1 mg/mL, [HEPES] = 5 mM, pH 7.0, [**1**] = 100 µM, T = 25 ^{o}C

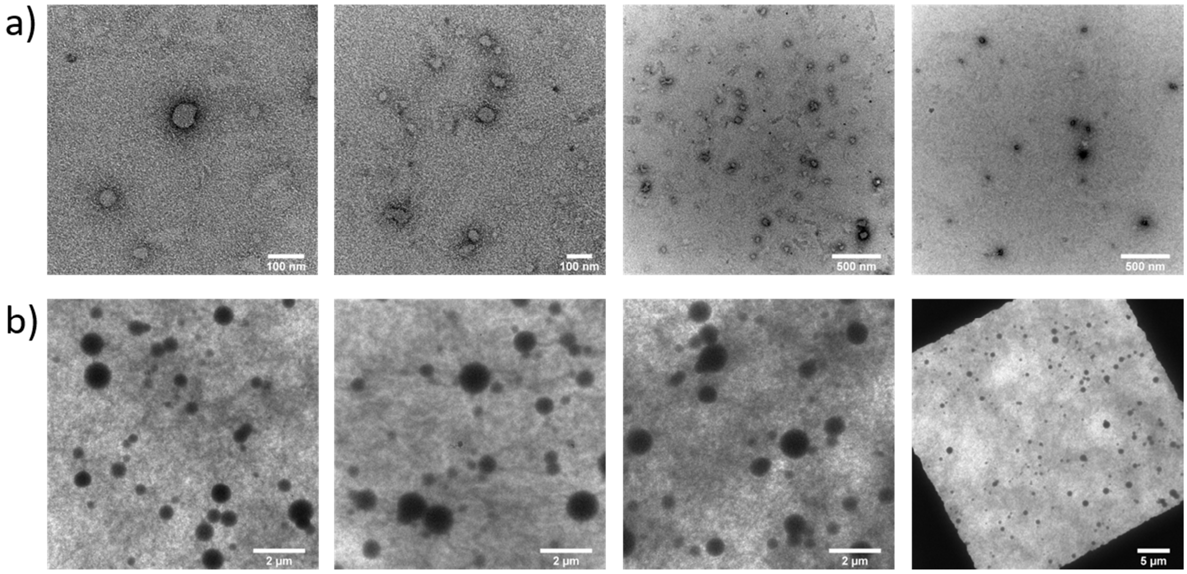

Figure S14. a) Additional TEM images of the assemblies formed in the transparent area (**I**) of the gel shown in Figure 4e. The sample was stained with uranyl acetate (2%) for 30 s. **b)** Additional TEM images of the assemblies formed in the opaque area (**II**) of the gel shown in Figure 4f. Gel compositions and experimental conditions: [agarose] = 1 mg/mL, [HEPES] = 5 mM, pH 7.0, [**1**] = 100 μM, T = 25 °C.

12. Laser scanning confocal microscopy (LSCM) images

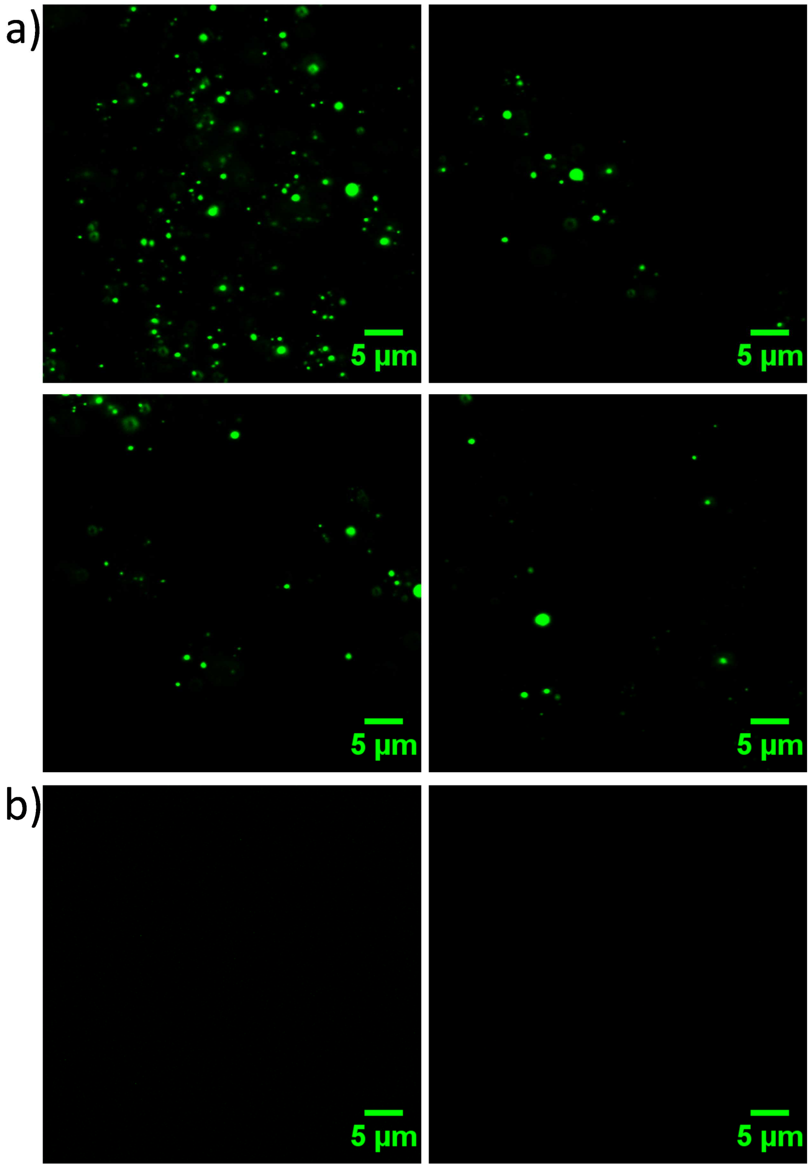

Figure S15. LSCM images of the gel to which 1 µL of ATP stock solution (1 mM) was injected in the center. **a)** Additional LCSM images from the opaque area in the gel center after 1 µL of a stock solution of ATP (1 mM) was injected at t = 300 min as described in the main text (Figure 2g). **b)** Additional LCSM images of the external transparent area from the gel to which 1 µL of a stock solution of ATP (1 mM) was injected in the gel center at t = 300 min as described in the main text (Figure 2i). Gel compositions and experimental conditions: [agarose] = 1 mg/mL, [HEPES] = 5 mM, pH 7.0, [**1**] = 100 µM, [Coumarin153] = 0.5 µM, T = 25 ^{o}C.

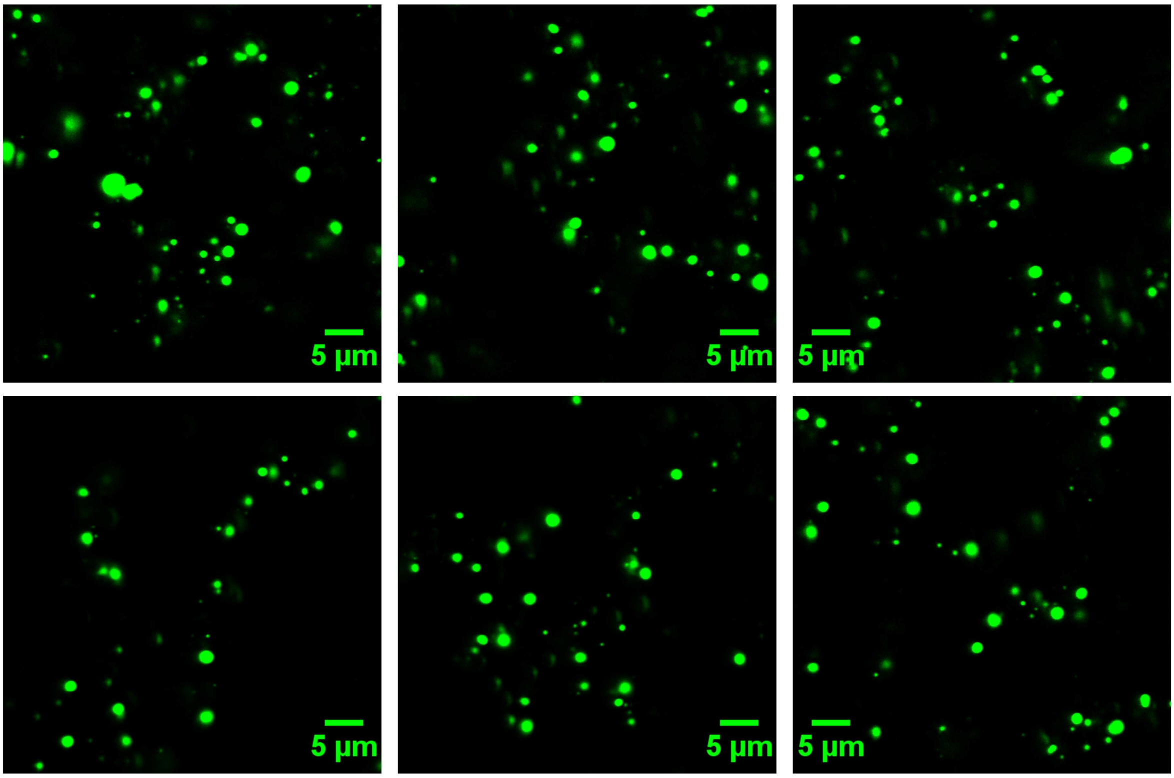

Figure S16. LSCM images of position 1 of a gel after addition of the 10th aliquot (1 μL) of an ATP stock solution (1 mM) at t = 8015 min as described in Figure 3g in the main text. Gel compositions and experimental conditions: [agarose] = 1 mg/mL, [HEPES] = 5 mM, pH 7.0, [**1**] = 100 μM, [Coumarin153] = 0.5 μM, T = 25 °C.

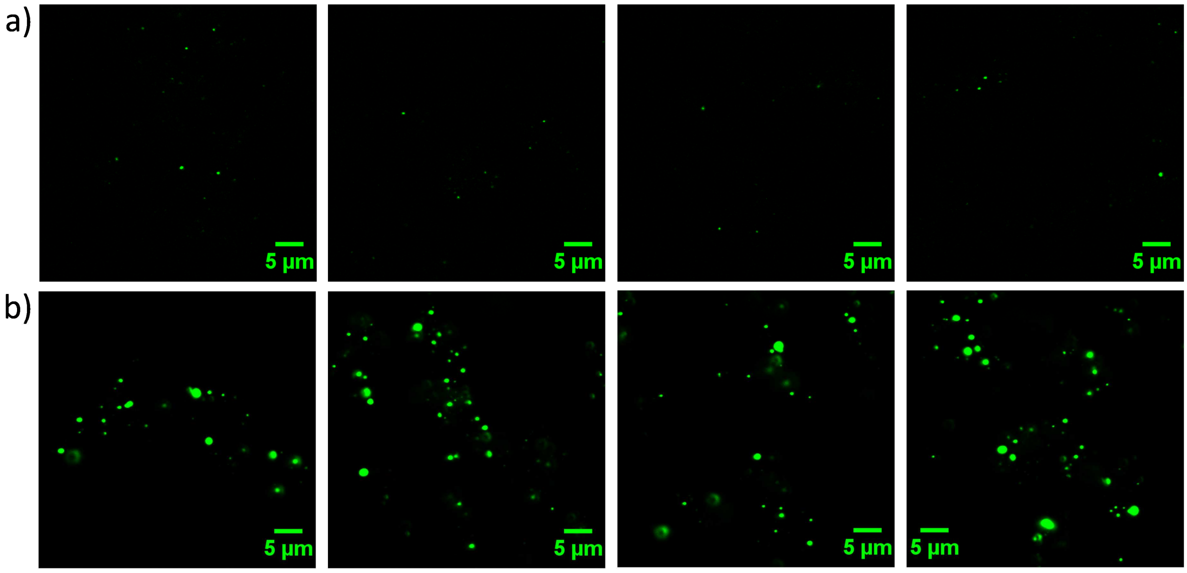

Figure S17. a) LSCM images of the assemblies formed in the transparent area (**I**) of the gel shown in Figure 4e. **b)** LSCM images of the assemblies formed in the opaque area (**II**) of the gel shown in Figure 4f. Gel compositions and experimental conditions: [agarose] = 1 mg/mL, [HEPES] = 5 mM, pH 7.0, [**1**] = 100 μM, [Coumarin153] = 0.5 μM, T = 25 °C.

13. Diffusion coefficients obtained from DOSY

Protocol for the preparation of the gel samples for DOSY measurements

A solution containing 3 mg agarose in D_2O (3 mL) was prepared and kept in a water bath at 40 °C. Aliquots (600 μL) of the solution were used to dissolve the compounds to be analysed by DOSY. The samples were sonicated at 40 °C to remove air bubbles. The DOSY experiments were carried out after the solutions had solidified to the gel-phase.

Diffusion coefficient of ATP

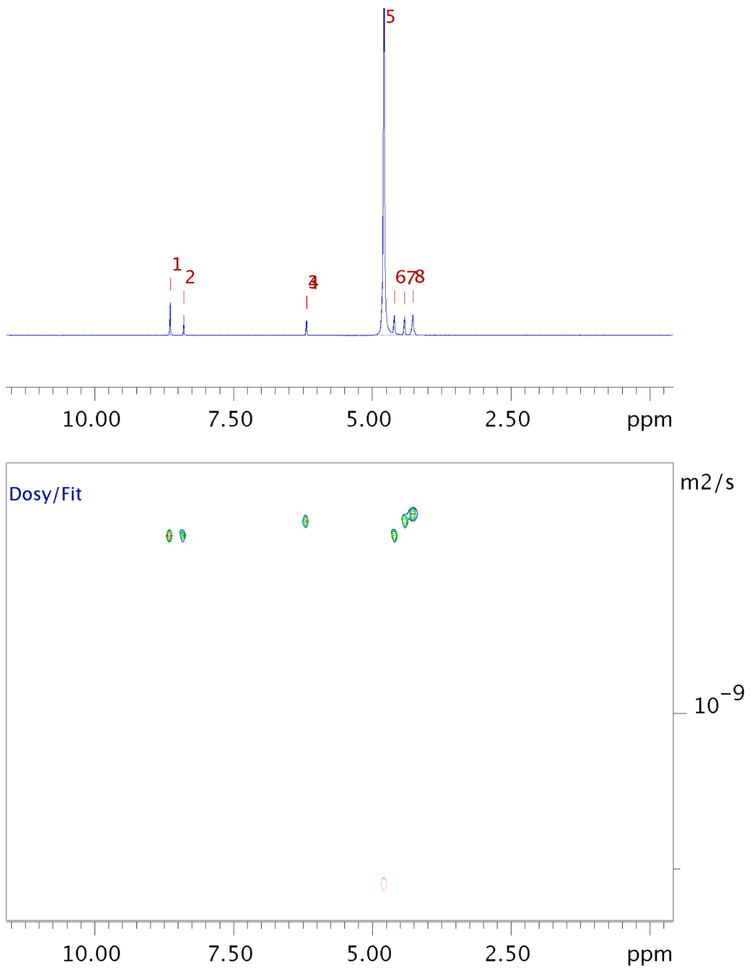

Figure S18. DOSY spectrum of ATP in D_2O (1 mM); D = (4.31 ± 0.16) × $10^{-10} m^2 s^{-1}$, 25 °C.

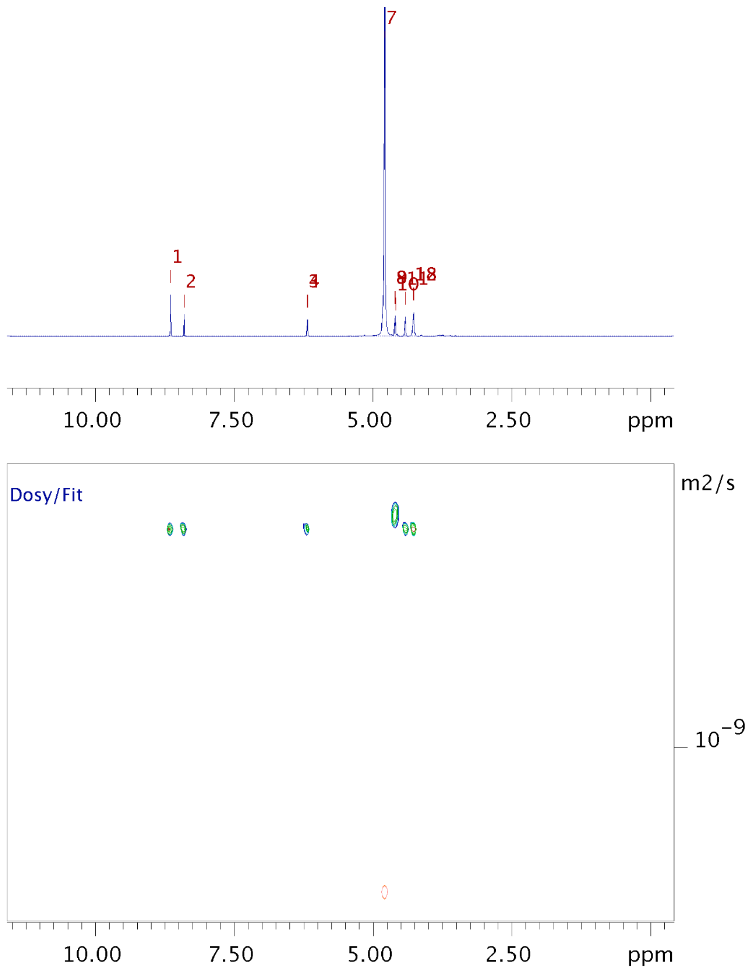

Figure S19. DOSY spectrum of ATP (1 mM) in agarose gel (1 mg/mL); D = (3.52 ± 0.11) × 10^{-10} m^2s^{-1}, 25 ^{o}C. Agarose was dissolved by D_2O.

Diffusion coefficient of 1

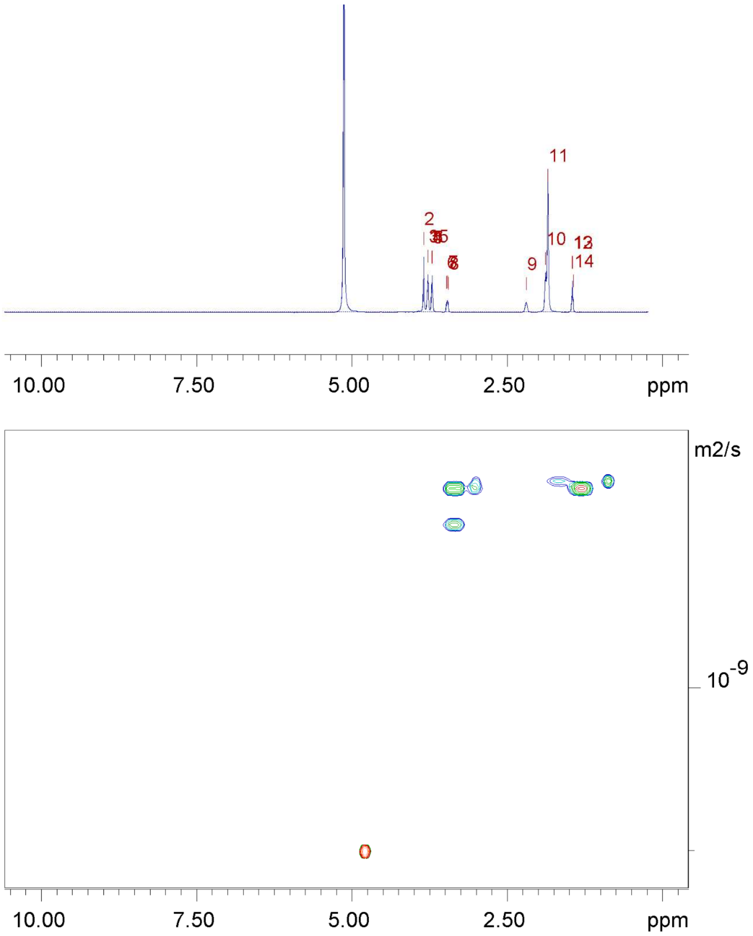

Figure S20. DOSY spectrum of $C_{12}TACN{\cdot}Zn^{2+}$ in D_2O (3 mM); D = (4.26 ± 0.20) × $10^{-10} m^2 s^{-1}$, 25 °C.

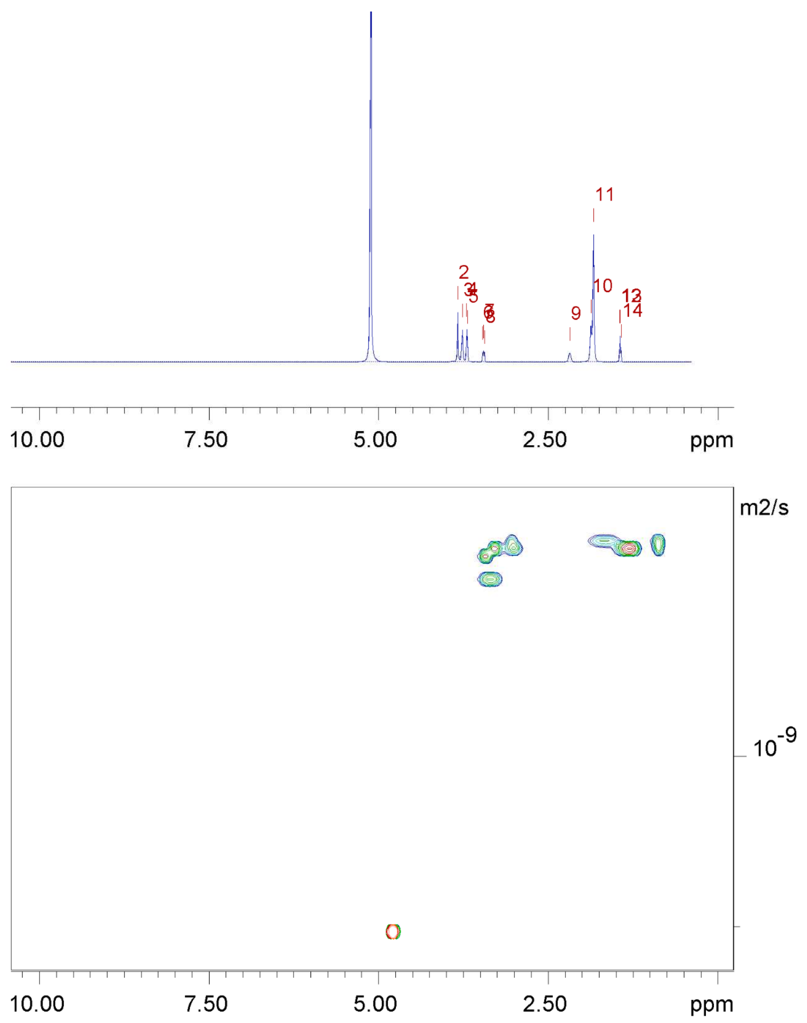

Figure S21. DOSY spectrum of $C_{12}TACN\cdot Zn^{2+}$ (3 mM) in agarose gel (1 mg/mL); D = (4.3 ± 0.2) × $10^{-10} m^2 s^{-1}$, 25 °C.

Because of precipitation it was not possible to carry out the DOSY experiments for a mixture of ATP (1 mM) and **1** (3 mM) in agarose gel or in D_2O.

14. UV-Vis spectra of compounds A, B and C in gel

The UV-Vis spectra of **A**, **B** and **C** in hydrogel show that **A** and **B** have no significant absorbance at 409 nm, which means that the absorbance increase at 409 nm can be taken as a reference for the formation of hydrazone **C**. The UV-Vis spectrum of hydrazone **C** is red-shifted and the absorption changed from a single peak with one maximum at 338 nm to a broad absorption band with three maxima at 366 nm, 384 nm and 408 nm (Figure S22a and S22b) in the presence of **1** and 30 μM/200 μM ATP both in solution and in gel. This can be interpreted as evidence for the change in environment as the hydrazone migrates from the aqueous buffer to the inner, more hydrophobic area inside the assemblies.

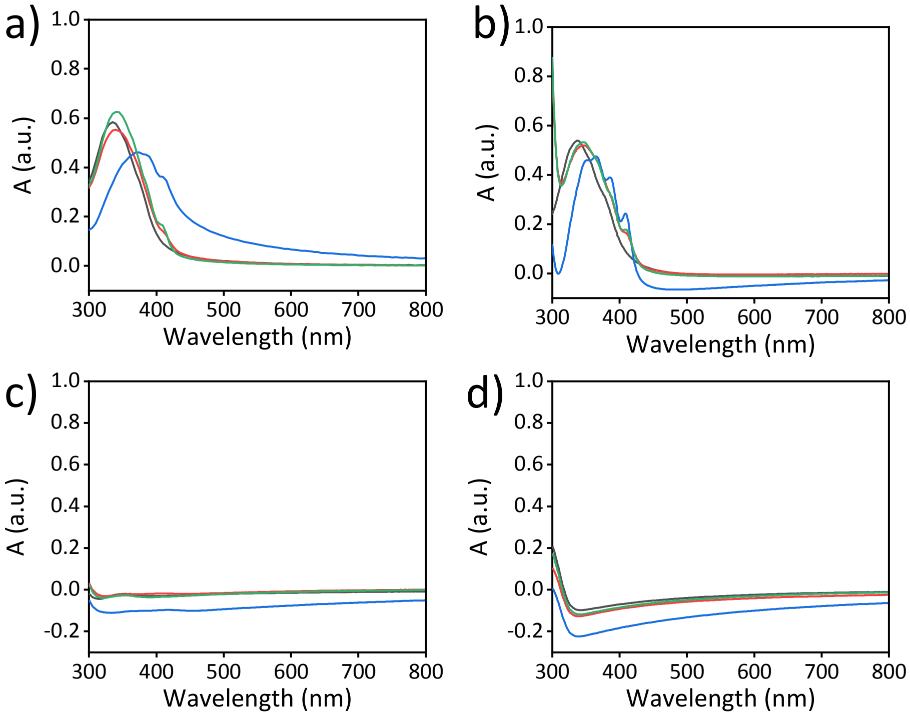

Figure S22. a) The UV-Vis spectra of 20 μM hydrazone **C** in solution under different conditions (see below for color codes). **b)** The UV-Vis spectra of 20 μM hydrazone **C** in gel under different conditions (see below for color codes). **c)** The UV-Vis spectra of 20 μM *trans*-cinnamaldehyde **A** in gel under different conditions (see below for color codes). **d)** The UV-Vis spectra of 20 μM 3-hydroxy-2-napthoic hydrazide **B** in gel under different conditions. Color codes: Black lines denote buffer, red lines denote buffer and **1** (100 μM), blue lines denote **1** (100 μM) and ATP (30 μM), green lines denote **1** (100 μM) and ATP (200 μM)). Gel composition and experimental conditions: [agarose] = 1 mg/mL, [HEPES] = 5 mM, pH 7.0, [**1**] = 100 μM, T = 25 ^{o}C.

15. UPLC analysis

Each sample for UPLC analysis is the combination of 6 gels from one microplate as shown in the photograph in FigureS21. For the sample taken from position 1 - where the reactions take place in the ATP-templated assemblies - at t = 24 h after ATP-injection the central turbid parts from 6 wells were collected in one vial (about 200 µL) and 100 µL methanol was added to dissolve the sample. In a similar way the sample from position 5 was prepared. Next, the samples were directly analysed by UPLC. The experiment was repeated in triple.

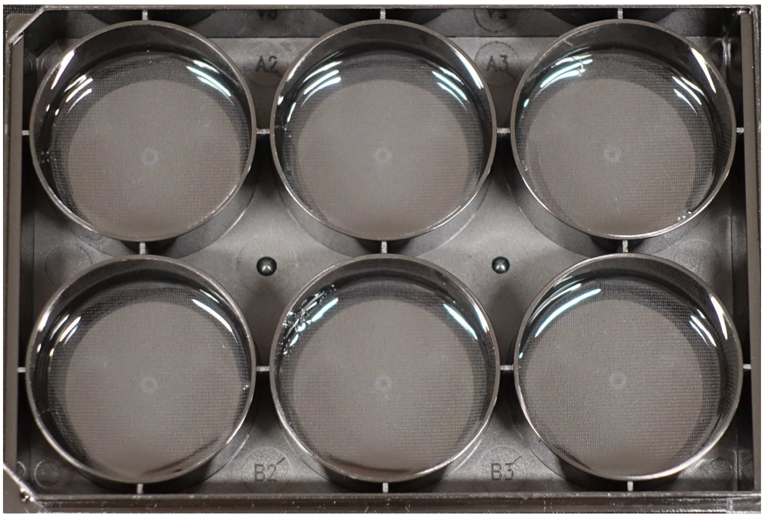

Figure S23. Photograph of the gel samples with 1 µL ATP (1 mM) injection in center as described in figure 5b in the main text.

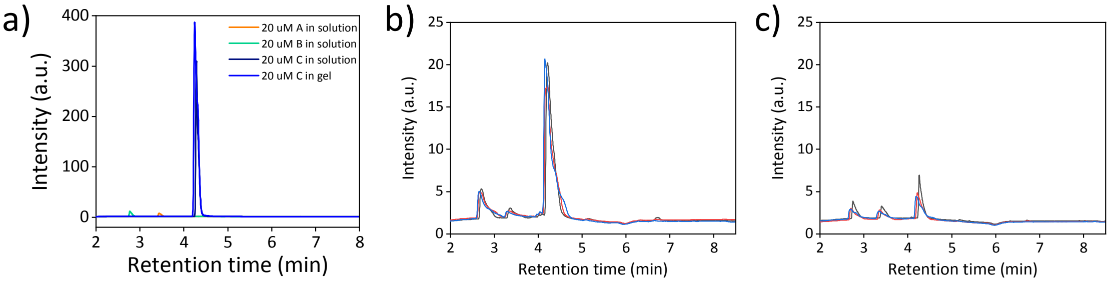

Figure S24. a) UPLC chromatograms of **A, B, C** monitored at 331 nm. Conditions in solution: [**A**]/[**B**]/[**C**] = 20 µM, [HEPES] = 5 mM, pH 7.0. Conditions in gel: [agarose] = 1 mg/mL, [HEPES] = 5 mM, pH 7.0, [**1**] = 100 µM, [ATP] = 35 µM. **b), c)** Three more sets of UPLC chromatograms of gel samples from position 1 and position 5 in the experiment described in figure 5c in the main text. All UPLC chromatograms monitored at 331 nm. Retention time: **A** = 3.4 min, **B** = 2.8 min, **C** = 4.2 min. Conditions: flow rate: 0.6 mL/min. gradient: 5-95% B (A: H_2O+0.1% HCOOH, B: ACN+0.1% HCOOH) from 0.00 to 5.00 min; 95-99.5 % B from 5 to 5.10 min; 99.5 % B from 5.10 to 8.0 min; 99.5-5 % B from 8.0 min to 8.2 min; and finally 5% B from 8.2 to 10.00 min. Injection volume: 20 µL. Column temperature: 50 ^{o}C.

16. Supplemental References

1. Cardona, M. A.; Prins, L. J. ATP-fuelled self-assembly to regulate chemical reactivity in the time domain. *Chem. Sci.* **2020**, *11* (6), 1518-1522.

2. Gasparini, G.; Martin, M.; Prins, L. J.; Scrimin, P. Limitations of the “tethering” strategy for the detection of a weak noncovalent interaction. *Chem. Commun.* **2007**, (13), 1340-1342.
